# Roles of bile acids signaling in neuromodulation under physiological and pathological conditions

**DOI:** 10.1186/s13578-023-01053-z

**Published:** 2023-06-12

**Authors:** Chen Xing, Xin Huang, Dongxue Wang, Dengjun Yu, Shaojun Hou, Haoran Cui, Lung Song

**Affiliations:** 1 Beijing Institute of Basic Medical Sciences, Taiping Road #27, Beijing, 100850 China; 2 College of Pharmacy, Jiamusi University, Jiamusi, 154007 China; 3 Anhui Medical University, Heifei, 230032 China

**Keywords:** Bile acids, Cholesterol, Blood brain barrier, FXR, TGR5, Neurodegenerative diseases, UDCA, TUDCA

## Abstract

Bile acids (BA) are important physiological molecules not only mediating nutrients absorption and metabolism in peripheral tissues, but exerting neuromodulation effect in the central nerve system (CNS). The catabolism of cholesterol to BA occurs predominantly in the liver by the classical and alternative pathways, or in the brain initiated by the neuronal-specific enzyme CYP46A1 mediated pathway. Circulating BA could cross the blood brain barrier (BBB) and reach the CNS through passive diffusion or BA transporters. Brain BA might trigger direct signal through activating membrane and nucleus receptors or affecting activation of neurotransmitter receptors. Peripheral BA may also provide the indirect signal to the CNS via farnesoid X receptor (FXR) dependent fibroblast growth factor 15/19 (FGF15/19) pathway or takeda G protein coupled receptor 5 (TGR5) dependent glucagon-like peptide-1 (GLP-1) pathway. Under pathological conditions, alterations in BA metabolites have been discovered as potential pathogenic contributors in multiple neurological disorders. Attractively, hydrophilic ursodeoxycholic acid (UDCA), especially tauroursodeoxycholic acid (TUDCA) can exert neuroprotective roles by attenuating neuroinflammation, apoptosis, oxidative or endoplasmic reticulum stress, which provides promising therapeutic effects for treatment of neurological diseases. This review summarizes recent findings highlighting the metabolism, crosstalk between brain and periphery, and neurological functions of BA to elucidate the important role of BA signaling in the brain under both physiological and pathological conditions.

## Introduction

Bile acids (BA), cholesterol-derived acidic steroids, are synthesized from cholesterol mainly in liver, stored in gallbladder and secreted into the gastrointestinal tract to absorb nutrients postprandially, efficiently reabsorbed from the intestine and finally returned to the liver. This cyclical movement is termed as the enterohepatic circulation helping to maintain BA homeostasis [1, 2]. BA not only mediate nutrients absorption, but also function as steroids hormones to regulate glucose, lipid and energy metabolism by binding and activating various nuclear receptors such as farnesoid X receptor (FXR) or membrane receptors such as takeda G-protein-coupled bile acid receptor 5 (TGR5) [1]. Importantly, BA presence in the brain under both physiological and pathological conditions attracts increasingly attentions on exploring the crosstalk of BA between periphery and central nervous system (CNS), and its roles in regulating brain function. Therefore, this review focuses on synthesis and communication of BA between the periphery and brain, physiological roles of BA in the CNS by direct or indirect pathways, and pathogenic or protective functions in neurological disorders.

## BA metabolism in periphery and brain

**Fig. 1:**
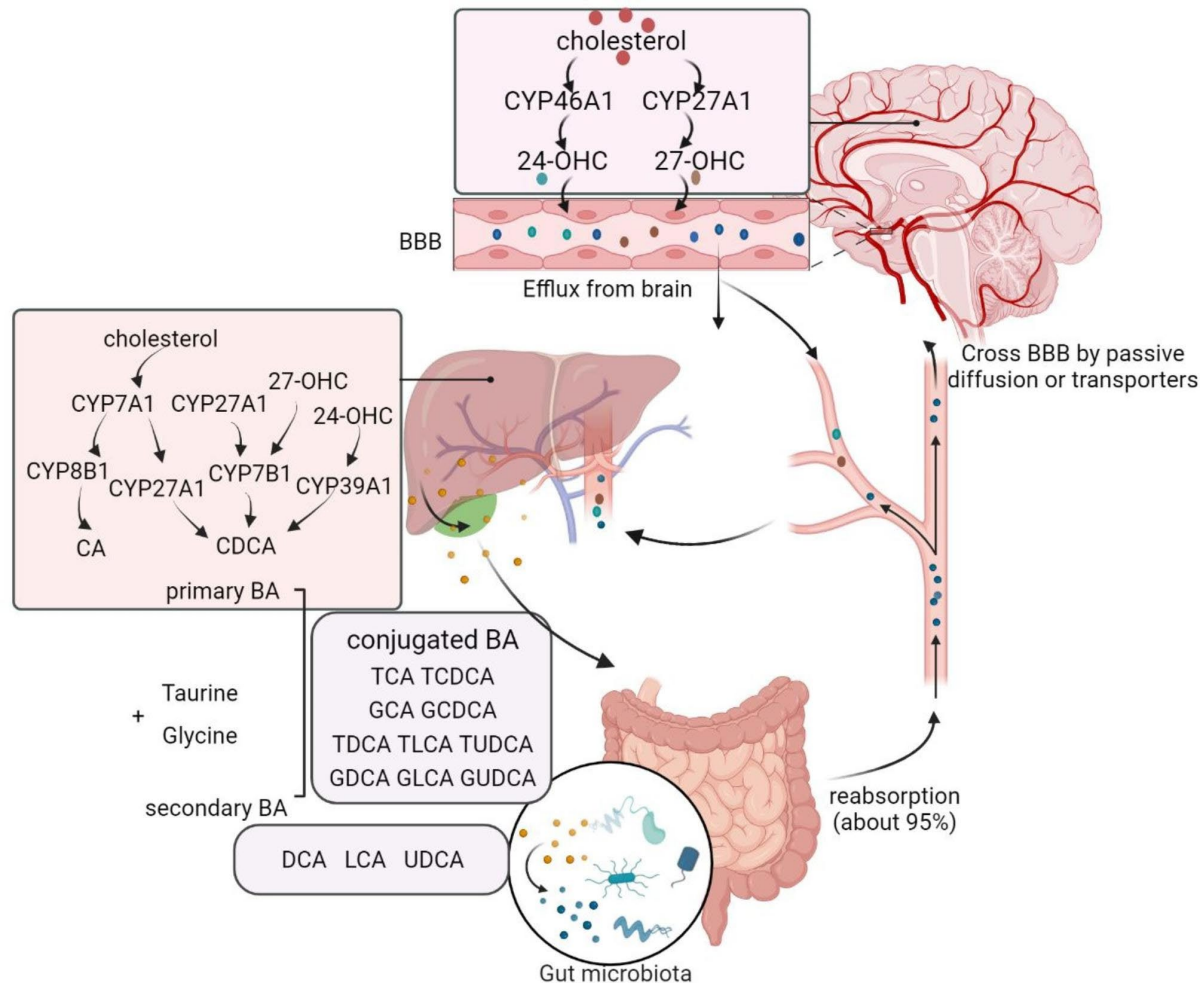
**Metabolism and circulation of bile acids in periphery and brain.** The catabolism of cholesterol to primary bile acids (BA) in liver by two pathways, involving the critical enzymes cholesterol 7α-hydroxylase (CYP7A1) and sterol 12α-hydroxylase (CYP8B1) in the classical pathway to form cholic acid (CA), and sterol 27-Hydroxylase (CYP27A1) and oxysterol 7α-hydroxylase (CYP7B1) in the alternative pathway to synthesize chenodeoxy cholic acid (CDCA). The neural cholesterol clearance pathway is initiated by CYP46A1 to form 24(S)-hydroxycholesterol (24-OHC) from cholesterol. 24-OHC subsequently crosses the BBB, and is metabolized by CYP39A1 in the liver to synthesize CDCA. The brain 27-OHC converted by CYP27A1 might also cross the BBB and is metabolized by CYP7B1 in the liver. The primary BA are converted to conjugated forms with glycine or taurine. Then, primary BA are further converted to the secondary BA metabolized by the gut microbes. After re-absorption through passive diffusion or bile acid transporters, about 95% of BA are reabsorbed in the ileum and returns to enterohepatic circulation. A small amount of BA in the systemic circulation can be taken up into the brain by passive diffusion or by active transport through the blood brain barrier (BBB).

The circulating BA pool composition consists of primary BA synthesized in the liver, and secondary BA transformed by specific gut microbiota. Additionally, the brain derived BA synthesis also contributes to the BA pool. There exists communication of circulating BA or the BA metabolic intermediates between periphery and brain by passive diffusion or BA transporters to cross the blood brain barrier (BBB). The main synthesis and circulation of BA in periphery and brain are summarized in Fig. 1.

### BA metabolism in liver and intestine

The catabolism of cholesterol to primary BA in periphery occurs predominantly in the liver by the classical pathway and the alternative pathway [3]. The classical pathway is initiated with the hydroxylation of cholesterol at the 7th position by CYP7A1 to generate 7α-hydroxycholesterol (7α-OHC). After epimerization of 7α-OHC via 3β-hydroxysteroid dehydrogenase type 7 (HSD3B7), the generated 7α-hydroxy-4-cholesten-3-one is either hydroxylated by CYP8B1 and then catalyzed by aldo-keto reductase family 1 member D1 (AKR1D1) and aldo-Keto reductase family 1 member C4 (AKR1C4) to produce 5β-cholestan-3α,7α,12α-triol, or catalyzed by AKR1D1 and AKR1C4 to generate 5β-cholestan-3α,7α-diol in the absence of CYP8B1. The steroid side chains of 5β-cholestan-3α,7α,12α-triol and 5β-cholestan-3α,7α-diol are then oxidized by sterol 27-hydroxylase (CYP27A1) to form cholic acid (CA) and chenodeoxycholic acid (CDCA), respectively [3]. The alternative pathway is initiated when cholesterol is hydroxylated at the 27th position by CYP27A1 to generate 27-hydroxycholesterol (27-OHC). Next, 27-OHC is further metabolized by CYP7B1 to generate 3β,7α-dihydroxy 5-cholestenoic acid, and then converted to CDCA. CA and CDCA are the two main primary BA. Especially, the majority of CDCA is converted to α-muricholic acid (α-MCA) and β-muricholic acid (β-MCA) in mice by cytochrome P450 2C70 (CYP2C70) [4]. By removing hydroxyl groups of CA and CDCA catalyzed by gut microbiota, deoxycholic acid (DCA) and lithocholic acid (LCA) are formed, respectively [5]. In mice, LCA can be further converted to ursodeoxycholic acid (UDCA) by 7a-hydroxylase, to hyodeoxycholic acid (HDCA) by 6a-hydroxylase, or to murideoxycholic acid (MDCA) by 6β-hydroxylase [6]. In humans, bacterial 7β-hydroxysteroid dehydrogenase (7β-HSDH) function to catalyze small amounts of CDCA to UDCA [6, 7]. Additionally, primary BA are converted to conjugated forms with glycine (mainly in humans) or taurine (mainly in mice) via bile acid-CoA synthase (BACS) and bile acid CoA:amino acid N-acyltransferase (BAAT) [1]. The conjugated BA contain taurocholic acid (TCA), glycocholic acid (GCA), taurochenodeoxycholic acid (TCDCA) and glycochenodeoxycholic acid (GCDCA). In mice, the generation of tauro-α-MCA (TαMCA), tauro-β-MCA (TβMCA) and tauroursodeoxycholic acid (TUDCA) also exists.

The conjugated BA could be transported by bile salt export protein (BSEP) and multidrug resistance associated protein 2 (MRP2) from the hepatocytes into the canaliculi, and subsequently the gallbladder to form the bile. After feeding or food intake, the presence of nutrients (especially fats and proteins) in the stomach triggers gallbladder emptying, leading to BA release into the duodenum. Then, BA are further converted to the major secondary BA metabolized by the gut microbes [8, 9]. The major secondary BA include deoxycholic acid (DCA), UDCA, lithocholic acid (LCA), hyocholic acid (HCA), hyodeoxycholic acid (HDCA), murideoxycholic acid (MDCA) and ω-muricholic acid (ω-MCA). Those unconjugated and some glycine-conjugated BA can be reabsorbed through passive diffusion in the jejunum and colon. And most of conjugated BA require active reabsorption via the apical sodium dependent bile acid transporter (ASBT) in the ileum. About 95% of BA are reabsorbed in the ileum and returns to the liver via sodium taurocholate co-transporting polypeptide (NTCP) and organic anion transport polypeptides (OATPs) mediated BA reuptake from the portal venous circulation [10–12]). About 5% of BA are excreted in feces. Thus, enterohepatic circulation of BA can maintain their own homeostasis by dynamical synthesis, transporting and reabsorption processes.

BA synthesis initiated by CYP7A1 in liver contributes to more than 70% of BA biosynthesis [13]. And, the alternative pathway initiated by CYP27A1 accounts for approximately 25–30% of synthesized BA in rodents but only about 5–10% BA pool in humans. Recently, it has been reported that BA concentrations in CYP7A1^−/−^CYP27A1^−/−^ mice showed reductions in plasma (45.9%), liver (60.2%), gallbladder (76.3%), small intestine (88.7%), and colon (93.6%) compared with the wide type mice [14]. And the female double knockout mice seemingly exhibit a large extend reduction in BA concentrations [15]. The reduction rather than completely abolished BA concentrations further provides the ideas that BA synthesis existing in other tissues also contributes to the BA homeostasis.

### Brain derived BA synthesis

Brain is the most cholesterol abundant organ containing about 20% of total amount of cholesterol [16]. Cholesterol is an essential component for cellular membrane, myelin, synapse and dendrite formation, a precursor of steroid hormones, and influence neuronal physiology in the development and adult stage [17]. Brain cholesterol is synthesized locally and independent of that in peripheral tissues due to BBB efficiently protecting the exchange of cholesterol with those in the circulation system. Noteworthy, the neural cholesterol clearance pathways by converting into BA provides the major excretion way in brain to balance the excess cholesterol.

Brain derived BA synthesis pathway requires the neuronal-specific and rate-limiting enzymes. Sterol 24-hydroxylase (CYP46A1), highly expressed in various brain regions such as the cerebral cortex, hippocampus, frontal lobe, caudate nucleus and amygdala, is located almost exclusively in the brain [18]. BA synthesis occurred in brain is through oxidation of cholesterol to 24(S)-hydroxycholesterol (24 S-OHC) catalyzed by CYP46A1. Unlike cholesterol, 24 S-OHC could pass through the BBB and rapidly diffuse into the systemic circulation for subsequent biotransformation by oxysterol 7a-hydroxylase II (CYP39A1) in the liver, leading to the synthesis of CDCA [19–22]. Therefore, the brain derived BA synthesis involves the initial stage of cholesterol clearance in brain, transport of 24 S-OHC from the BBB to the periphery and the final synthesis in liver.

Though the contribution of CYP46A1 mediated pathway to the overall BA synthesis is minor, the conversion of cholesterol into 24 S-OHC is the most important cholesterol clearance pathway in the CNS. CYP46A1 deficiency could impair 24 S-OHC synthesis in the CNS, leading to the reduced brain and serum 24 S-OHC concentrations, and deficits in the cognitive function including spatial, associative, and motor learning [23, 24]. Another study also has shown that knockdown of CYP46A1 expression in the striatum reproduced the Huntington’s disease phenotype with spontaneous striatal neuron degeneration and motor deficits [25]. However, knockout of CYP46A1 gene in mice caused about just 40% reduction in brain cholesterol excretion, suggesting other routes of cholesterol removal from the brain independent of CYP46A1 existing as well [24].

CYP27A1 expression exists in almost all cells in the body. There is a constant flux of 27-OHC formed by CYP27A1 and metabolites of this oxysterol from extra-hepatic sources to the liver for further conversion into BA. CYP27A1 and CYP7B1 are also expressed in the brain, might suggesting that the primary BA can also be synthesized in brain [26–28]. By utilizing a mouse model with over-expressed CYP27A1, the levels of 27-OHC increased about 12-fold in the brain [28]. 27-OHC can cross the BBB and is rapidly eliminated from the brain and metabolized in the liver. Another study also found a novel metabolic route for the elimination of 27-OHC in the brain to be metabolized into a steroid acid, 7α-hydroxy-3-oxo-4-cholestenoic acid, which could further cross the BBB and enter into the circulation [29]. The *CYP27A1*^*−/−*^ mice showed the reduced pool size of BA, and increased cholestanol in both plasma and brain [30, 31]. Accordingly, the expression of CYP27A1 in brain seems to function as another way to remove cholesterol. Additionally, CYP7B1 in the brain is involved in neurosteroid metabolism by catalyzing hydroxylation of 27-OHC, dehydroepiandrosterone and pregnenolone [19, 32, 33]. Taken together, function of CYP27A1 and CYP8B1 in brain is likely to remove cholesterol, but whether they contribute to BA synthesis in brain remain to be further confirmed.

## The crosstalk of BA between CNS and periphery

The continuous flow of information between the brain and peripheral tissues is important in maintaining physiological functions. Especially, circulating BA metabolites acting as a communication bridge between the gut and the brain have been attracting increasingly attentions. In the gut lumen, the dihydroxylation of primary BAs is driven by a limited number of microbes belonging to the firmicutes phylum, and subsequent de-conjugation of glycine or taurine from the sterol core of conjugated BAs is catalyzed by several microbial species including Lactobacilli, Clostridium, Bifidobacteria and Bacteroides expressing functional enzymes bile salt hydrolases (BSHs) [34]. Additionally, some other BA transformations such as oxidation/reduction reactions and hydroxyl groups epimerization can also be catalyzed by gut microbiota including Actinobacteria, Proteobacteria, Firmicutes, Bacteroidetes, Clostridium, Ruminococcus and Eubacterium species [35, 36]. The microbiota-encoded transformations of primary BA finally generate secondary BA, contributing to the intestinal BA composition and total BA pool size [37, 38]. With the emerging evidences of crucial connections between the gut microbiota and the brain function or neurological disorders, the microbiota metabolites BA are possible to function as an important communication channel in the gut-brain axis [39, 40].

Recently, the presence of multiple primary and secondary BA metabolites is found in the brain under physiological condition. Studies have proved that various bile acids species including both conjugated and unconjugated BA are found in brain metabolomic profiles of both humans and rodents [41–44]. In an early study, three protein-bound unconjugated BA including CA, CDCA and DCA were identified in the rat brain cytoplasmic fraction [41]. Using high-sensitivity and high-resolution mass spectrometry, a panel of 20 bile acids has been identified in the rat brain metabolome, most of which have not previously been documented [43]. By comparing BA compositions and levels in the brain and plasma of both human and mouse samples, different BA profiles have been identified in brain tissues including the common BAs (CA, LCA, TCA and DCA), human special BAs (CDCA, GCA, GCDCA, GDCA, TCDCA, UDCA), and mouse special BAs (TUDCA, β-MCA and Ω-MCA) [44]. The identified BA metabolites in brain contain both primary BA synthesized in the liver and secondary BA converted in intestinal by microbiota. Therefore, there should be direct crosstalk or communication of BA between CNS and periphery.

The brain BA pool can directly originate from the systemic circulation. It has been suggested that unconjugated BA could be transported across the BBB and reach the brain at low concentrations by passive diffusion depending on their hydrophobic properties [41, 45, 46]). For example, UDCA showed the ability to penetrate across experimental blood-brain barrier model by using confluent monolayer of human brain microvascular endothelial cells in a time-dependent manner [47]. Brain levels of CA, CDCA and DCA positively correlated with serum levels, and intraperitoneally injected deuterium-labeled CA and CDCA were well-detected in rat brain [48]. For conjugated BA, the conjugation of BA with glycine or taurine can increase the amphipathicity and solubility, which makes them impermeable to cell membranes. Various BA transporters have been gradually found to be expressed in the CNS including MRP2, NTCP and BSEP in the choroid plexus [49], OATP in brain capillary endothelial cells [50], ASBT in the hypothalamus [51, 52], providing a mechanism for the neuronal uptake of conjugated BA from the periphery into the CNS by BA transporters. But, direct evidence of in vivo BA transport to cross the BBB via their transporters is still lacking. Recently, it is reported that several endogenous BA, mainly the tauro-conjugated species such as TDCA, TCA, TCDCA, TαMCA and TβMCA, could rapidly reach the hypothalamus and transiently increase in 30 to 60 min after physiological feeding at the beginning of the dark phase of mice [53]. Similarly, orally administrated BA mix containing the sodium salt of taurocholic, glycocholic, deoxycholic and cholic acids also can rapidly reach the hypothalamus [53]. Altogether, BA could be transported across the BBB and reach the brain by passive diffusion or transporters uptake, which correlates with their unconjugated or conjugated format.

The BBB integrity and permeability are sensitive to BA concentration, which could be modified or even damaged by increased concentration of BA exposure. Abnormal high concentrations of sodium deoxycholate and taurochenodeoxycholate acting as strong detergents could damage the lipid layers of the BBB, while a slightly increased concentrations may modify the BBB permeability in a more subtle way [54]. Additionally, CDCA and DCA could disrupt endothelial tight junctions by increasing occludin phosphorylation in a rac1-dependent manner, and lead to increased permeability of the BBB [55]. Therefore, peripheral BA can more easily reach the brain due to the increased BA compositions in circulation system or disturbed BBB permeability under pathological conditions, which is consistent with multiple reported abnormal BA profiles in brain tissues or cerebrospinal fluid (CSF) in neurological disorders [56].

## The physiological functions of BA in CNS

It has been widely reported that BA act as signaling molecules in non-nervous systems by activating various nuclear or membrane receptors. FXR and TGR5 are two of the most studied BA receptors. Besides, other receptors are also activated by BA, such as pregnane X receptor (PXR), vitamin D receptor (VDR), liver X receptor (LXR), glucocorticoid receptor (GR) and sphingosine-1-phosphate receptor 2 receptor (S1PR2) [57]. Though the above receptors are also found in the brain, whether BA induce neurological functions directly through above receptors in brain remains to be elucidated. Functional FXR was recently found in mouse and human brains [58, 59]. By using the FXR knockout mice, deficiency of FXR signaling disturbed the balance of GABA to Glu in the hippocampus and cerebellum, and impaired cognitive function and motor coordination [60]. However, the direct evidences to verify BA activated brain FXR signaling in regulating brain function still need to be further explored. Recently, another study has proved that postprandial BA can reach the brain and control satiety in response to physiological feeding [53]. Peripheral or central administration of a BA mix or a TGR5-specific BA mimetic (INT-777) could exert an anorexigenic effect via activating TGR5 in orexigenic agouti-related peptide/neuropeptide Y (AGRP/NPY) neurons in the hypothalamic arcuate nucleus [53].

Some studies also find that neurological functions of BA are generated through affecting activity of the ligand-gated ion channels. BA can regulate neural excitability, proliferation, and differentiation by affecting the functions of neurotransmitter receptors such as N-methyl-D-aspartate (NMDA) receptor and γ-aminobutyric acid (GABA) type A (GABAA) receptor. The NMDA receptor, an ionotropic glutamate receptor, plays important roles in synaptic communication by causing Ca2^+^ influx and activating calmodulin to trigger CaMKII, MAPK, CREB, and PI3K pathways [61]. The GABAA receptor is a ligand-gated chloride ion channel, whose activation causes an influx of chloride ions and results in subsequent neuron hyperpolarization and neurotransmission inhibition [62]. Endogenous neurosteroids are potent modulators of NMDA receptor and GABAA receptor [63, 64]. Similarly, CDCA and CA were able to block GABAA and NMDA receptors, and CDCA was more powerful to reduce the firing of hypothalamic neurons and synchronize the network activity by antagonizing the NMDA receptor and GABAA receptor [65]. GABA functions to induce sleep by binding GABAA receptor expressed in histaminergic neurons and suppressing activation of the histaminergic neurons in the tuberomammillary nucleus (TMN) of the hypothalamus [66, 67]. UDCA could block GABAA receptor on TMN neurons to mediate disinhibition of the histaminergic system, functioning to promote wakefulness during the active period of the day in wild type mice but not in histamine-deficient mice [68]. Moreover, TUDCA was proved to enhance the proliferation, self-renewal, and neuronal conversion of neural stem cells (NSCs) [69]. Adult neurogenesis occurs in two specific regions including the subgranular zone (SGZ) of the hippocampal dentate gyrus (DG) and the subventricular zone (SVZ) of the lateral ventricles. TUDCA could impact on postnatal NSC fate by selectively inducing SVZ-derived NSCs proliferation and neural differentiation but not DG-derived NSCs [70]. And, intracerebroventricular administration of TUDCA in adult rats markedly enhanced both NSCs proliferation and early differentiation in SVZ regions [70]. Thus, the above findings support that BA exerts the neurological functions by neurotransmitter receptors expressed in local brain regions.

In addition to the direct effects triggered by BA in brain, peripheral BA may also provide the indirect signal to the CNS via FXR dependent fibroblast growth factor (FGF)-15/19 pathway or TGR5 dependent glucagon-like peptide-1 (GLP-1) pathway [71]. Mouse FGF-15 and its human orthologue FGF19 are hormone-like enterokines mainly produced by the ileum under the transcriptional control of BA-activated FXR. In the intestine, BA absorbed by enterocytes can activate the nuclear receptor FXR and lead to the production of FGF15/19 [72]. The enterocytes release FGF15/19 into the portal vein to exert the roles in inhibiting BA synthesis or regulate the metabolism of lipids and glucose [73]. Importantly, FGF19 in the systemic circulation can cross the BBB and is relatively stable in the brain [74]. The expression of fibroblast growth factor receptor (FGFR) 4, the main target receptors of FGF15/19, has been detected in the hypothalamus and medial habenular nucleus [75, 76]. Interestingly, the orexigenic AGRP/NPY neurons in the hypothalamic arcuate nucleus responds to FGF19 administration by both intraperitoneal and intracerebroventricular injection in mice [77]. The central effects of FGF19 reduces hypothalamic AGRP/NPY neuron activity and improves peripheral glucose homeostasis [75, 77]. Another study has proved that TCA gavage could increase FGF15 transcriptional expression in the ileum and improve oral glucose tolerance in obese mice. And, the mice with FGFR1 deficiency specifically in AGRP/NPY neurons lose the responsiveness to TCA, suggesting that FGFR1 is necessary for mediating the beneficial effects of TCA on glucose tolerance [78]. Taken together, FGF15/19 signaling in the CNS is closely associated with energy and glucose tolerance. However, the critical question remains whether increase of circulating FGF15/19 induced by intestine BA is sufficient to elicit a substantial effect in the CNS under normal physiological conditions.

In the intestine, TGR5 activation by BA in entero-endocrine L-cells can result in the production and release of the gut hormone GLP-1, which is capable of transmitting BA signal from the intestine to other parts of the body [79–81]. Intestinal GLP-1 may reach the brain via the systemic circulation or through the vagal nerve [82–85]. The GLP-1 receptor is expressed in various tissues including the CNS and vagal neurons [86, 87]. GLP-1 is capable of cross the BBB or transmit signal via vagal nerve afferents from the gastrointestinal tract to the CNS [88, 89]. Via the vagal-brainstem-hypothalamic pathway, peripheral GLP-1 can affect many brain regions and consequently exerts its inhibitory effect on food intake and increased perception of satiety [85, 90–93]. Noteworthy, GLP-1 could be rapidly inactivated by the enzyme dipeptidyl peptidase IV (DDP-4), which is expressed in the endothelial membranes of the capillaries and present in the plasma. Thus, it is more likely that intestine BA may signal to the brain via GLP-1 pathway transmitted through vagal nerve rather than cross the BBB via the systemic circulation [94]. However, the exact implications and contribution of BA for this signaling route remains to be elucidated in future research.

**Fig. 2:**
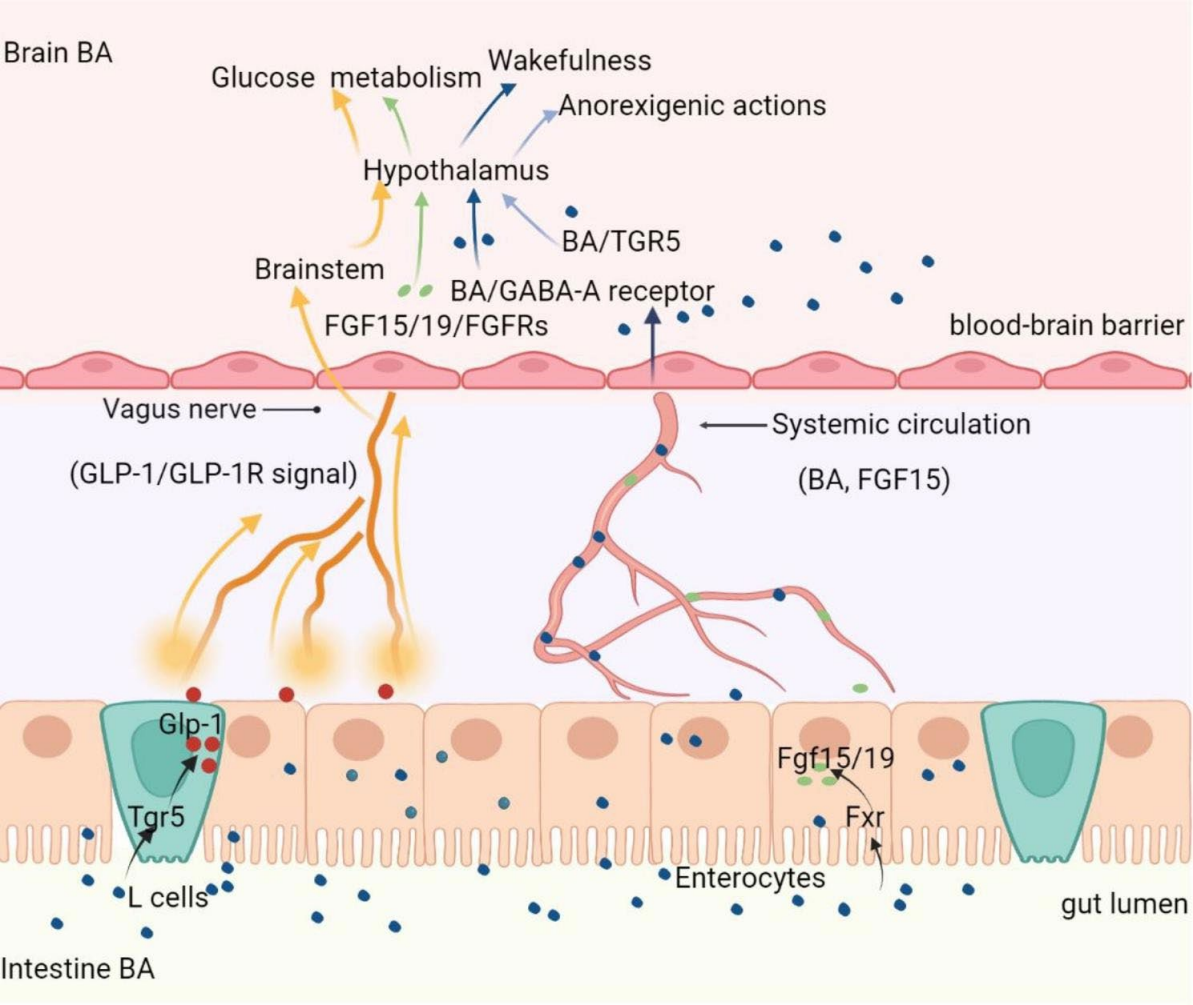
**Bile acids signal to the central nervous system (CNS).** Bile acids (BA) in the intestinal lumen can signal to the CNS via the direct pathway or indirect pathway. Bile acids in the intestine escape the enterohepatic circulation, reach the systemic circulation, and cross the blood-brain barrier (BBB) to interact with receptors in the brain. BA in the CNS could exert an anorexigenic effect via activating TGR5 in the hypothalamic arcuate nucleus or block GABAA receptor on tuberomammillary nucleus (TMN) of the hypothalamus to promote wakefulness. BA taken up by enterocytes can activate the nuclear receptor FXR to promote FGF15/19 production. FGF15/19 is released by the enterocytes, enters the systemic circulation, and cross the BBB to interact with FGF receptors in the brain. The central effects of FGF15/19 are involved in glucose metabolism and energy homeostasis. TGR5 activation by BA in enteroendocrine L-cells triggers GLP-1 production. GLP-1 could interact with GLP-1 receptors expressed on afferent terminals of the vagal nerve present in the lamina propria and portal vein. The vagal nerve projects to the brainstem, from where projections are further directed toward other brain regions. GLP-1 is believed to exert its inhibitory effect on food intake and energy homeostasis via the vagal-brainstem-hypothalamic pathway

In general, BA may exert neuromodulation effect in the CNS via either activating membrane and nucleus receptors, or affecting the functions of neurotransmitter receptors. Periphery BA communicates with the CNS via the systemic circulation directly reaching the brain or indirect pathway mediated by FXR-FGF15/19 pathway or TGR5-GLP-1 pathway. A summary of pathways of BA signal to CNS can be seen in Fig. 2.

## Functions of BA in neurological disorders

The cytotoxic property of BA critically correlates with their hydrophobicity. The magnitude of hydrophobicity of unconjugated BA would be UDCA < CA < CDCA < DCA < LCA. By conjugating with glycine or taurine, BA become more hydrophilic amidated forms. The retention of hydrophobic BA can cause injury of hepatocyte, which involves in disrupting cell membranes, promoting reactive oxygen species (ROS) production, causing mitochondrial dysfunction, inducing endoplasmic reticulum stress, and leading to apoptosis or necrosis [95]. Noteworthy, the hydrophilic BA, UDCA and its taurine-conjugated derivative TUDCA, are widely concerned for their low toxicity and the protective properties against cholestatic liver diseases by improving the antioxidant activity and inhibiting apoptosis. Recently, the variation of BA metabolites in the diseased brains also illustrates that BA correlate with the pathogenesis of various neurological diseases (Table 1), or act as neuroprotective factors to improve symptoms in some neurological diseases (Table 2).

**Table 1:** The altered brain BA metabolites in neurological diseases

Neurological diseases	BA metabolites in brain
HE	Increased brain BA in CSF (GUDCA and GCA) of HE patients, and total BA contents especially TCA and LCA in HE models.
AD	Lower brain TCA, higher primary BA (GCDCA or TMCA) and secondary BA (DCA, LCA, GDCA, GLCA, GUDCA, TDCA or TLCA), increased the ratios of secondary versus primary BA in brain (DCA:CA, TDCA:CA, GDCA:CA, GDCA:DCA, TLCA:CDCA or GLCA:CDCA) in AD patients. Decreased brain CA, TCA, TMCA, β-MCA, Ω-MCA, or TUDCA in AD APP/PS1 mice model.
PD	Identified affected BA metabolites (increased DCA, MCA and decreased TCA and GCDCA) in CSF of sporadic PD or genetically predisposed (LRRK2) to PD patients.
HD	Decreased brain CYP46A1 expression and reduced the intermediates 24 S-OHC in HD patients or HD mice models.
CTX	Accumulated cholesterol and cholestenol (CDCA precursor).

**Table 2:** The neuroprotective role of UDCA/TUDCA in neurological diseases

Protective roles	Neurological diseases
Inhibits neurotoxic (A1) polarization and activation of astrocytes.	AD, PD, MS
Inhibits microglial activation, decrease pro-inflammatory mediators (TNFa, IL-1β, IL-6, HMGB1, IFN-γ, IFN-β, NO, iNOS, COX-2) production, increase anti-inflammatory factors (IL-10, IL-4, TGF-β, ANXA1) production.	Acute neuroinflammation, AD, PD, HD, MS, SCI, RP
Inhibit apoptosis of neurons via suppressing cytochrome c release, decreasing caspases activation (caspases 2, 3, 6, 12), inhibiting E2F-1/p53/Bax apoptotic pathway, triggering a PI3K dependent survival signaling pathway, or promoting MR nuclear translocation and transactivation.	AD, PD, HD, SCI, ALS
Prevent oxidative stress in neurons via modulating activation of AMPK, JNK and AKT, increasing the expression of antioxidant factors (Nrf2, DJ-1, HO-1, GPx, parkin), preventing ATP levels decrease and ROS production, improving mitochondrial stabilization and function.	PD
Inhibits endoplasmic reticulum stress prevent tau hyperphosphorylation via UPR inhibition, reduce aberrant conformational conversion	AD, Prion diseases

### Altered BA metabolites in neurological diseases: the potential pathogenic factors

Hepatic encephalopathy (HE), a serious complication of both chronic liver disease and acute liver failure, manifests brain dysfunction characterized with a wide spectrum of neurological or psychiatric abnormalities ranging from mild cognitive impairment to marked disorientation, confusion, and coma [96]. Serum BA are known to increase in conditions where the liver is damaged in acute and chronic liver disorders. Importantly, altered BA profiles in brain or cerebrospinal fluid (CSF) also have been verified in HE patients and experimental models. The blood concentrations of total BA and especially the conjugated BA were substantially increased in cirrhotic patients with HE compared to cirrhotic patients without HE or the healthy controls [97]. BA levels in CSF showed a great increase for GUDCA (93-fold) and GCA (241-fold) in HE patients [98]. In the brains of rats with HE induced by bile-duct ligation, the toxic LCA was extremely higher concentration (87.4% of total brain BA) while LCA was absent in controls [99]. By using azoxymethane (AOM) injection induced HE mice model, increased total BA content in brain tissue also has been demonstrated [59]. And TCA was identified to be specifically increased in cortex of AOM-induced HE mice [100]. Furthermore, via the administration of BA sequestrant cholestyramine to AOM-induced HE mice, the circulatory and brain BA levels were decreased, and neurological decline was delayed [59]. Conversely, CA or DCA feeding worsened AOM-induced neurological decline, suggesting that BA species contribute to the neurological decline in HE [59]. Studies have revealed that mechanisms by which BA triggering neurological decline in HE involved in both FXR and S1PR2 signaling [59, 100]. Specifically, they found that activation of cortical S1PR2 by increased TCA levels triggered microglial activation and consequent neuroinflammation in the AOM-treated mice [100]. Together, the findings suggest the elevated BA pool size in the brain acts as a possible systemic pathogenic contributor to brain dysfunction in HE.

Alzheimer’s disease (AD), an aggressive degenerative disease leading to dementia in old age, characterize with neuropathological changes including aggregated beta-amyloid (Abeta) plaques and neurofibrillary tangles, astrogliosis, microglial activation and massive neuronal demise [101]. Currently, the altered BA compositions are increasingly concerned in both experimental models and clinical mild cognitive impairment or AD patients. In plasma of amnestic mild cognitive impairment patients or AD patients, the levels of some BA including DCA, LCA, GDCA or GUDCA have been reported to be significantly increased when compared with the age-matched control subjects [102–104]. Another study also reported the significantly lower concentration of serum CA and brain TCA in AD patients compared to cognitively normal older adults [44]. In the APP/PS1 mice model of AD, the levels and compositions of brain BA are also likely to be disturbed during the development of AD pathology. Higher CA in serum, higher LCA and lower TMCA in brain were detected in 6 months old APP/PS1 mice. While, levels of brain CA, TMCA, β-MCA, Ω-MCA, TCA, and TUDCA were decreased in 12 months old APP/PS1 mice [44]. Noteworthy, the altered BA metabolites and the ratios of secondary BA versus primary BA are furtherly found to be associated with brain structural and functional impairment in AD. Based on the brain transcriptomics and metabolomics of postmortem AD samples, higher ratio of GCDCA/CA and secondary BA (DCA, LCA, TDCA, and GDCA) were identified in AD patients compared with cognitively normal individuals [42]. Similarly, increased levels of secondary BA (DCA, GDCA, and TDCA) in serum, and an increased DCA:CA ratio in serum and brain was found to be closely associated with cognitive decline [105]. By using targeted metabolomic profiling, the association of serum-based BA metabolites with neurodegeneration biomarkers and structural changes of AD were identified [106]. Higher GDCA:CA levels was associated with greater amyloid deposition. Higher levels of primary GCDCA, secondary GLCA and TLCA was correlated with higher CSF p-tau values. Increased levels of primary GCDCA, secondary BA (GLCA, TLCA) and the ratios of secondary BA to primary BA (TDCA:CA, GDCA:CA and GLCA:CDCA) were significantly associated with reduced cortical thickness and hippocampal volume. Altogether, the findings provide a new perspective to show that increased levels of secondary cytotoxic BAs and their ratios to primary BA are closely associated with the pathogenesis of AD. The presence of secondary BA in the brain also strongly indicates the important role of gut-brain axis and gut microbiome in physiological changes of AD. Collectively, the current findings support the idea that circulating and brain BAs may function as a contributor to AD pathogenesis.

Parkinson’s disease (PD), the second most common neurodegenerative disease manifesting the progressive motor deterioration, is characterized with death of dopaminergic neurons and the accumulation of intracytoplasmic α-synuclein-containing Lewy bodies in the substantia nigra [107]. In studies of both clinical PD patients and surgical rodent models of PD, alteration of BA metabolism is observed. An observed elevation of unconjugated BA (CA and DCA) as well as conjugated BA (TDCA, GDCA) were found in plasma of PD patients compared to health control, which also could be alleviated by levodopa treatment [107]. BA analysis in PD patients also revealed an increase in appendix (DCA and LCA) and in ileum (LCA) relative to the controls [108]. By metabolic profiling of CSF from people suffering sporadic PD and genetically predisposed (LRRK2) to PD, BA metabolism was also identified as one of the major affected biochemical pathways, especially the aberrant concentration of GDCA, TCA and GCDCA [109]. Utilizing rodent PD model by stereotactic unilateral injection of human α-synuclein fibrils, three BA including (MCA-Ω, TUDCA and UDCA) were found to be down-regulated in serum of prodromal phase of PD mice compared with the controls [110]. By using the concentration of three BA combined with logistic regression, the prodromal PD mice could be discriminated from control mice with high accuracy following cross validation. The data highlights the potential of BA as biomarkers of predicting PD in the early stage and possibly reflecting the progression of PD.

Huntington’s disease (HD), an inherited autosomal-dominant neurodegenerative disease, manifests the motor, behavioral and cognitive dysfunctions in clinical symptoms [111]. The expansion of the CAG repeats in exon 1 of the huntingtin gene on chromosome four can induce neuronal loss in the striatum and cause synaptic dysfunction. Currently, the clinical and rodent based animal researches still lack direct evidences on studying the association of BA profiles with HD pathogenesis. However, recent findings suggest that altered cholesterol homeostasis may contribute to the pathophysiology of HD [112]. Although BA levels have not been described in brain and circulation system of HD, the expression of CYP46A1 and level of the metabolite 24 S-OHC have been found to be reduced in the plasma of HD patients [113, 114] as well as in the brain of several yeast artificial chromosome (YAC) mice (carrying progressively increased CAG repeats) and knock-in mouse models [113–115]. As was reported, CYP46A1 expression is decreased in the putamen of HD patients, in the striatum of R6/2 mice (a HD mouse model), and in striatal Huntington’s disease cell lines (by transfection of mutant huntingtin gene) [25]. CYP46A1 knockdown in the mouse striatum induced a decrease of 24 S-OHC levels and produced a spontaneous striatal neurodegeneration associated to abnormal balance and motor coordination. While, CYP46A1 restoration in the striatum of R6/2 mice increased 24 S-OHC levels, decreased neuronal atrophy and improved motor deficits. The study provides the evidence that the neuroprotective role of CYP46A1 in Huntington’s disease. Together, these data suggest that the impaired brain derived BA synthesis may contribute to HD pathology.

Cerebrotendinous xanthomatosis (CTX), a rare neurodegenerative disorder caused by autosomal recessive mutations in the CYP27A1 gene, exhibits infantile-onset diarrhoea, juvenile-onset cataracts, young adult-onset tendon xanthomas, progressive neurological dysfunction and so on [116, 117]. The neurological progression includes most common corticospinal tract abnormalities (weakness, hyperreflexia, spasticity, ataxia, cognitive decline, and gait difficulty) and less common abnormalities (seizures, psychiatric and speech changes) [118]. Deficiency of CYP27A1 leads to impaired the ability to convert cholesterol into CDCA, and causes the accumulation of cholesterol and cholestenol (CDCA precursors) in tendon, artery and brain [119]. Though BA metabolites in brain of CTX is rarely concerned, BA replacement therapy by CA and especially CDCA administration are used to reduce the accumulated plasma cholestanol and improve clinical symptoms in CTX [120, 121]. However, to improve the neurological symptoms of CTX, the age at diagnosis and initiation of CDCA treatment closely correlates with the prognosis [122, 123]. Though the molecular mechanisms underlying the effects of CDCA on the neuropathological phenotypes in CTX patients remains unclear, the impaired BA synthesis and reduced CDCA largely contribute to CTX pathology and neurological dysfunction.

### Function of BA in treatment of neurological diseases: the neuroprotective roles

UDCA and its taurine-conjugated derivative TUDCA are regarded as the most hydrophilic BA [124]. Presently, UDCA is the only drug approved by the US FDA for the treatment of primary biliary cirrhosis [38, 39]. Attractively, the use of UDCA or TUDCA as an agent to treat various neurological diseases is now a promising consideration by triggering anti-neuroinflammatory response, inhibiting the apoptosis, reducing oxidative stress, protecting mitochondria or might acting as a chaperone to correct misfolded proteins.

#### The anti-neuroinflammation effects

Neuroinflammation occurs to harmful stimuli and injuries during infections, traumatic injury, or neurological diseases. It is characterized with activation and proliferation of microglia and astrocytes in the CNS, and followed by morphological changes and release of proinflammatory mediators [125, 126]. Increasing studies have provided the convincing evidences of neuroinflammation in contributing to the pathogenesis of neurological disorders [127].

UDCA can exert the anti-inflammation effects by suppressing the microglial activation and pro-inflammatory cytokines production. In an early study, UDCA effectively inhibited the pro-inflammatory interleukin-1 beta (IL-1β) and nitric oxide (NO) induced by beta-amyloid 42 (Abeta42) or lipopolysaccharide (LPS) in rat microglial cells [128]. Further, UDCA were found to inhibit the degradation of IkappaB to block expression of the NF-kappaB dependent genes responsible for inflammation in Abeta42 treated BV-2 microglia cells [129]. Thus, UDCA functions to attenuate the production of pro-inflammatory mediators via inactivation of NF-kappaB in microglia to inhibit neuroinflammation. Additionally, in 1-methyl-4-phenyl-1,2,3,6-tetrahydropyridine (MPTP) induced mouse model of PD, UDCA treatment decreased levels of tumor necrosis factor α (TNF-α), interleukin-6 (IL-6), IL-1β and interferon-γ (IFN-γ) and prevented MPTP-induced degeneration of DA neurons, whose mechanism correlates with the inhibition of phosphorylation of JNK and p38MAPK to against neuroinflammation [130].

The anti-inflammatory property of TUDCA also has been verified in various pathological conditions. In acute neuroinflammation mice model, TUDCA specifically reduced microglial activation in the hippocampus, expressions of monocyte chemotactic protein-1 (MCP-1) and vascular cell adhesion 1 (VCAM-1) required for microglial migration and blood monocyte invasion to the CNS inflammation site [131]. The reported molecular basis of TUDCA exerting the anti-inflammatory effect was involved in reducing nitrite production by transcriptional inhibition of inducible nitric oxide synthase (iNOS), and inhibiting pro-inflammatory stimuli-induced NF-kappaB activation in glial cells [131]. Furthermore, TUDCA presented an additional anti-inflammatory mechanism through activation of transforming growth factor β (TGFβ) pathway in mouse brain [132]. Another study also proved that TUDCA biased the microglial phenotype toward the anti-inflammatory both in vivo and in vitro. By binding to the bile salt receptor GPBAR1/TGR5, TUDCA caused an increase in intracellular cAMP levels in microglia to induce anti-inflammatory markers, while reducing pro-inflammatory ones [133]. Similarly, TUDCA treatment efficiently alleviated behavioral impairments, reversed TGR5 down-regulation, attenuated microglia activation, prevented neuroinflammation via inhibiting NF-kB signaling to decrease pro-inflammatory cytokines (IL-1β, TNFα, IL-6) and increase anti-inflammatory cytokine (IL-10) in the hippocampus [134].

TUDCA also showed promising efficiency in weakening the secondary neuroinflammatory caused by the primary trauma injury. Spinal cord injury (SCI), a type of severe trauma or injury to the spinal cord, is characterized with complete or incomplete spinal motor and sensory dysfunction [135]. Due to the irreversible process of the primary injury caused by initial mechanical damage, the subsequent secondary injury caused by ischemia and edema needs to be greatly concerned and solved. Resident microglia and hematogenous macrophages are key inducers to provoke the deleterious inflammatory response after SCI. Recently, TUDCA based anti-neuroinflammatory therapy also shows promising efficiency in SCI treatment. TUDCA was able to decrease inflammatory mediator including NO, TNF-α, IL-1β, cyclooxygenase-2 (COX-2), and iNOS in LPS-stimulated BV2 microglial cells, RAW 264.7 macrophages and bone marrow-derived macrophages (BMDMs) [136]. Simultaneously, in SCI rat model, TUDCA treatment supported the recovery of the injury site and contributed to the polarization of macrophages/microglia toward M2 type to reduce inflammatory reaction by suppressing the expression of inflammatory factors (iNOS, CD68 and CD86) [136, 137]. TUDCA was also proved to attenuate PKM2 pathway activation in proinflammatory microglia and inhibited the expression of IL-6, IFN-β and high mobility group protein 1 (HMGB1) in lesion center [138]. The treatment of injectable hydrogel containing TUDCA also significantly decreased inflammatory cytokine levels (TNF-α, IL-1β, IL-6, IFN-γ) and suppressed the phosphorylation of extracellular signal-regulated kinase (ERK) and c-Jun N-terminal kinase (JNK) and p38 in the mitogen-activated protein kinase (MAPK) pathway in injured spinal cord segments [139]. TUDCA was also proved to promote the differentiation of BMDMs derived M2 macrophages. And, transplantation of TUDCA-induced M2 macrophages in rat SCI model decreased pro-inflammatory cytokines (IL-1β, TNF-α and IL-6) and increased the anti-inflammatory cytokine (IL-4), which correlates with the inhibition of phosphorylation of the ERK, JNK and p38 in the MAPK signal pathway [140]. However, a recent study provided the evidences that TUDCA treatment only reduced neuroinflammation and improved recovery of autonomic and motor functions in the subacute phase of SCI, but not support long term functional recovery in the chronic phase [141]. Together, TUDCA seemingly shows partial promising therapeutical value in weakening the secondary neuroinflammatory caused by the primary trauma injury.

TUDCA could alleviate the chronic neuroinflammation by inhibiting astrocytosis or microglia activation in neurodegenerative diseases including AD, PD, multiple sclerosis (MS) and retinitis pigmentosa. In AD, amyloid deposition leads to extensive microgliosis and astrocytosis surrounding the affected areas. It has been described that TUDCA supplementation could mitigate the activation of glial cells in APP/PS1 mice [142]. Similarly, another study also showed that TUDCA treatment ameliorated astrocytosis and microgliosis in the hippocampus and frontal cortex in APP/PS1 mice, as well as the production of proinflammatory cytokines especially TNF-α [143]. In MPTP induced PD mice model, TUDCA inhibited both astrocyte and microglia activation [144, 145]. And, TUDCA attenuated IL-1β expression and increased the anti-inflammatory protein ANXA1 expression in the cortex [145]. In the chronic demyelinating MS, lower levels of circulating BA metabolites were found in multiple cohorts of adult and pediatric patients with MS compared with controls by using metabolomic profiling [146, 147]. More importantly, TUDCA supplementation blocked neurotoxic polarization of astrocytes and proinflammatory polarization of microglia, reduced innate immune cell infiltration and the severity of behavioral and pathological changes in an animal experimental autoimmune encephalomyelitis model of MS, which results in amelioration of neuroinflammation through the effects of TUDCA on GPBAR1 [147]. In inherited neurodegenerative retinitis pigmentosa model, the beneficial effects of TUDCA resulted from its anti-inflammatory activity by reducing numbers and activation of microglial cells [148]. TUDCA was also capable to enhance phagocytosis of photoreceptors outer segments by retinal pigment epithelium cells via the activation of Mer tyrosine kinase (MerTK) receptor activation [149]. Taken together, TUDCA treatment shows efficiency in alleviating chronic neuroinflammation in neurodegenerative disorders.

#### The anti-apoptotic effects

Studies on animal models and cell lines reveal that apoptosis seems to play a crucial role in the progression of some neurological disorders [150]. The anti-apoptotic effects of UDCA or TUDCA have been extensively studied and characterized in vitro studies on cultured neuronal cells and in vivo studies on animal diseases models.

The hallmarks of AD pathological changes are involved in amyloid plaque deposits and fibrillary tangles. The protective effects of UDCA and TUDCA in inhibiting apoptosis have been verified in vitro studies on amyloid-β treated neuronal cells and in vivo studies on transgenic AD murine models. In vitro studies, the apoptosis induced by Abeta incubation in primary rat neurons or differentiated rat neuronal-like PC12 cells could be prevented by UDCA or TUDCA [151–153]. Similarly, the anti-apoptotic effects of TUDCA were also seen in vitro model of familial AD by expressing APP with the Swedish mutation, or double-mutated human APP and PS1 in mouse neuroblastoma cells [154]. Furtherly, the perturbation of mitochondrial apoptotic pathway was found to be a crucial event in UDCA or TUDCA mediated inhibition of Abeta induced apoptosis. UDCA and, more efficiently, TUDCA treatment abolished Abeta induced mitochondrial membrane permeabilization and subsequent cytochrome c release in isolated neuronal mitochondria. And, Abeta exposure-driven perturbation of mitochondria structural changes, including the mitochondrial membrane redox status, the lipid polarity and disrupted protein mobility, could be almost completely abolished by TUDCA treatment [155]. Importantly, the anti-apoptotic property of TUDCA could be exerted by interfering with upstream signal of mitochondria apoptosis. TUDCA could abrogate Abeta induced apoptosis through inhibiting c-Jun N-terminal kinase (JNK) nuclear localization and caspase-2 activation, interfering an E2F-1/p53/Bax apoptotic pathway, reducing nuclear fragmentation and the activity of caspases 2 and 6 and modulating activity of p53, Bcl-2 and Bax, or triggering a phosphatidylinositol 3-kinase (PI3K) dependent survival signaling pathway [152–154, 156]). One study also identified that TUDCA promoted mineralocorticoid receptor (MR) dissociation from its cytosolic chaperone hsp90 and subsequent MR nuclear translocation and transactivation to prevent Abeta induced neuronal apoptosis [157]. Besides, TUDCA has also been shown to alleviate the toxic downstream effects of Abeta by inhibiting the levels of apoptosis and caspase-3 activation, and abolishing the caspase-3 cleavage of tau into a toxic species in primary rat cortical neurons [158]. Considering studies in vivo, TUDCA supplementation could attenuate amyloidogenic amyloid precursor protein (APP) processing, reduce Abeta deposition in hippocampal and prefrontal, and prevent the spatial, recognition and contextual memory defects in APP/PS1 mice [142, 159].

The main characteristic of PD is the progressive death of dopaminergic neurons in substantia nigra region of the brain. The application of TUDCA decreased the number of apoptotic cells and facilitated the survival of DA neurons in serum-free condition in vitro. Similarly, TUDCA pretreatment could improve the survival and function of nigral transplants in a rat model of PD [160].

In mouse models of HD, TUDCA has been shown to suppress cytochrome c release, decrease caspases activation, inhibit DNA fragmentation and reduce the number and volume of striatal lesions, resulting in enhanced neuronal survival and improved sensorimotor and locomotor deficits. In 3-nitropropionic acid (3-NP) induced HD model, TUDCA significantly reduced 3-NP-mediated cell death of cultured striatal neurons, as well as apoptosis and lesion in 3-NP administrated rats [161]. In the R6/2 transgenic mouse model of HD, systemically administration of TUDCA led to reduced striatal atrophy, decreased striatal apoptosis, fewer and smaller size ubiquitinated neuronal intranuclear huntingtin inclusions, and improved locomotor and sensorimotor ability [162].

In SCI, apoptosis is an important event of the secondary injury after initial trauma damage. TUDCA could reduce the injury degree and suppress apoptosis via up-regulating anti-apoptotic factor Bcl-2 or inhibiting the expression of pro-apoptotic factors (Bax, caspase-3, caspase-12) [163, 164]. The possible mechanisms of TUDCA in suppressing apoptosis might involve in the activation of autophagy by increasing expression of autophagic related proteins (Beclin-1,LC3II/I) or inhibition of the ER stress via decreasing ER stress-related factors (IRE1, Chop, ATF6, Grp78 and Erdj4 ) [165–168].

Amyotrophic lateral sclerosis (ALS), the most common adult-onset motor neuron disease, is selectively progressive deterioration and loss of function of the motor neurons in the brain and spinal cord [169]. Gene mutations such as superoxide dismutase 1 (SOD1), chromosome 9 open reading frame 72 (C9orf72) and so on are associated with ALS pathogenesis. After well absorbed, UDCA could cross the BBB and slow the rate of progression in ALS patients [170, 171]. Similarly, a slower progression in ALS patients and delayed muscle denervation in an hSOD1^G93A^ mouse model of ALS were also observed after TUDCA administration [172, 173]. But the efficiency and especially potential mechanism of UDCA or TUDCA still needs substantial evidences in future. Interestingly, GUDCA, glycine-conjugated UDAC, also seemingly functioned to inhibit apoptosis in a commonly used model of ALS in vitro using the motor neuron-like NSC-34 cells carrying the hSOD1^G93A^ mutation. GUDCA treatment prevented changes in mitochondria dynamic properties and apoptosis by reducing caspase-9 levels, which indicating the promising anti-apoptotic effects of GUDCA to slow disease onset and progression of ALS [174].

#### The anti-oxidative effects

The mitochondrial dysfunction and oxidative stress are strongly implicated in PD, and the anti-oxidative role of TUDCA are mainly studied in PD. As was reported, TUDCA treatment prevented MPTP-induced decrease of dopaminergic fibers, ATP levels and mitochondrial function [175]. And, TUDCA improved mitochondrial number and function by preventing 1-methyl-4-phenylpyridinium (MPP+)-induced ROS production and ATP depletion in mouse cortical neurons. Moreover, TUDCA could exert its neuroprotective role in a parkin-dependent manner in contributing to mitochondrial stabilization and modulation of mitophagy to protect from mitochondrial dysfunction and oxidative stress [176]. These observations indicated that the clearance of dysfunctional mitochondria can be an important antioxidant and pro-survival strategy of TUDCA in PD treatment.

Nuclear factor erythroid 2 related factor 2 (Nrf2), a master regulator of cellular redox status, is responsible for activating the transcription of antioxidant enzymes glutathione peroxidase (GPx) and heme oxygenase-1 (HO-1). TUDCA could protect from both MPP + and α-synuclein-induced oxidative stress by activating Nrf2 in human neuroblastoma SH-SY5Y cells. Additionally, the anti-oxidative stress effect of TUDCA was further evaluated in PD mice mode treated with MPTP In vivo. TUDCA pre-treatment prevents the deleterious effects of MPTP by preventing the decrease in ATP levels, promoting AMPK activation, and sustaining increased levels of antioxidant enzyme HO-1 and parkin [145]. It was observed that TUDCA treatment increased the expression of Nrf2, Nrf2 stabilizer DJ-1, and antioxidant enzymes HO-1 and GPx [177]. Particularly, TUDCA efficiently protected against dopaminergic cell loss in the nigrostriatal axis in MPTP-induced neurodegeneration PD mouse model, which involves in the impairment of ROS production, modulation of JNK activity and activation of the AKT pro-survival pathway [178].

#### The inhibition of endoplasmic reticulum stress

Endoplasmic reticulum (ER) stress is characterized with the accumulation of unfolded or misfolded proteins in the ER lumen, and the initiation of a cascade of signal transduction called unfolded protein response (UPR). TUDCA could act as a molecular chaperone to ameliorate ER stress and preventing UPR dysfunction in ER stress related diseases such as hepatobiliary disorders, diabetes and obesity [179]. Interestingly, UPR is also activated in neurodegenerative tauopathies such as AD. TUDCA has been shown to prevent tau hyperphosphorylation via inhibition of the UPR in human neuroblastoma cell lines [180]. Moreover, TUDCA administration to a transgenic mouse model of familial amyloidotic polyneuropathy was able to reduce transthyretin toxic aggregates, in turn decreasing apoptotic and oxidative biomarkers that are usually associated with transthyretin deposition [181]. The results support the inhibition of UPR as a possible mechanism underlying the neuroprotective actions of TUDCA in AD.

Prion diseases, a class of rare and incurable neurodegenerative diseases, are characterized with aberrant conformational conversion and aggregation of cellular prion protein (PrP^C^) into the disease-associated ‘scrapie’ isoform (PrP^Sc^) [182]. UDCA/TUDCA treatment has been used to target and delay protein aggregation plaguing prion diseases, but the efficiency shows complex consequences. One study have provided the evidences for aggregation inhibitory effects and neuroprotective roles of TUDCA and UDCA in prion disease [183]. For example, TUDCA and UDCA substantially reduced PrP conversion in chronically and acutely infected cell cultures and decreased neuronal loss in prion-infected cerebellar slice cultures. Low dose UDCA treatment in early stage could prolong incubation periods, reduce astrocytosis and increase survival time in prion-infected male mice but not in female mice [183]. Similarly, low dose TUDCA treatment in chow 7 days post inoculation showed protective effects by increasing incubation periods and significantly increased phosphorylated eIF2α levels. However, treatment with high-dose TUDCA or UDCA provides no therapeutic benefit. The delayed treatment with high-dose UDCA is ineffective and could even worsen outcomes [184]. Currently, the overall misfolded protein-diminishing or ER stress-alleviating activity of UDCA/TUDCA is still unclear and lack of enough substantial evidences in prion diseases and other neuropathological conditions.

## Conclusions and remarks

This review summarizes recent findings to highlight the crosstalk of BA between brain and periphery, and neurological functions of BA under both physiological and pathological conditions. Under physiological circumstances, the presence of BA metabolites, as well as the receptors and transporters support the direct neurological functions of BA in the brain. BA function as important steroid hormones to exert neuromodulation effect in the CNS through either activating membrane and nucleus receptors or affecting the functions of neurotransmitter receptors. The crosstalk of BA between periphery and brain might communicate via the systemic circulation directly reaching the brain or indirect pathway mediated by FXR-FGF15/19 pathway or TGR5-GLP-1 pathway. However, the substantial effects directly triggered by BA in brain, especially postprandial increase of circulating BA, remain to be explored. Considering the indirect pathway, BA contribute to the production of FGF15/19 by activating FXR in enterocytes or the production of GLP-1 through activating TGR5 in enteroendocrine L-cells in the intestine. But more studies are also required to confirm whether increase of plasma FGF15/19 is sufficient to exert substantial effect through the presence of FGFRs in the CNS. Due to the high level of postprandial GLP-1 in the lamina propria of the intestine, the TGR5-GLP-1 pathway is more likely to signal to the CNS via the vagal nerve present in the lamina propria. However, the exact implications and contribution of BA for this signaling route in CNS remains an interesting subject for future research.

BA act as etiological factors or mediators linking the pathogenesis of various neurological diseases, supporting BA metabolites as potential risk biomarkers for prognosis of diseases. Due to the complex of BA metabolites in compositions and biochemical properties, how to figure out the exact function of specific BA metabolite or the overall contribution of altered BA profiles to the pathogenesis of neurological diseases remains to be solved in the future. Attractively, the supplementation of hydrophilic UDCA and TUDCA have been proved to display therapeutic benefits by inhibiting the neuroinflammatory response, apoptosis, oxidative stress, protecting mitochondria or even acting as a potential chaperone to correct misfolded proteins in various neurological diseases. The neuroprotective role of UDCA, especially TUDCA provides promising strategies for treatment of neurological diseases. Currently, the prevalence of BA in neurological research is a growing field. Better understanding of BA signaling and its effects in the brain will provide novel discoveries and strategies for controlling these debilitating diseases in future.

## Data Availability

Not applicable.

## Abbreviation

BA: Bile acids
FXR: Farnesoid X receptor
TGR5: Takeda G-protein-coupled bile acid receptor 5
CNS: Central nervous system
CYP7A1: Cholesterol 7α-hydroxylase
CYP8B1: Sterol 12α-hydroxylase
CYP27A1: Sterol 27-Hydroxylase
CYP7B1: Oxysterol 7α-hydroxylase
HSD3B7: 3β-hydroxysteroid dehydrogenase type 7
CA: Cholic acid
CDCA: Chenodeoxycholic acid
AKR1D1: Aldo-keto reductase family 1 member D1
27-OHC: 27-Hydroxycholesterol
UDCA: Ursodeoxycholic acid
α-MCA: α-muricholic acid
β-MCA: β-muricholic acid
CYP2C70: Cytochrome P450 2C70
BACS: Bile acid-CoA synthase
BAAT: Bile acid CoA:amino acid N-acyltransferase
TCA: Taurocholic acid
GCA: Glycocholic acid
TCDCA: Taurochenodeoxycholic acid
GCDCA: Glycochenodeoxycholic acid
TαMCA: Tauro-α-MCA
TβMCA: Tauro-β-MCA
TUDCA: Tauroursodeoxycholic acid
BSEP: Bile salt export protein
MRP2: Multidrug resistance associated protein 2
DCA: Deoxycholic acid
LCA: Lithocholic acid
HCA: Hyocholic acid
HDCA: Hyodeoxycholic acid
MDCA: Murideoxycholic acid
ω-MCA: ω-muricholic acid
ASBT: Apical sodium dependent bile acid transporter
NTCP: Sodium taurocholate co-transporting polypeptide
OATPs: Organic anion transport polypeptides
BBB: Blood brain barrier
CYP46A1: Sterol 24-hydroxylase
24S-OHC: 24(S)-hydroxycholesterol
CYP39A1: Oxysterol 7α-hydroxylase II
CSF: Cerebrospinal fluid
PXR: Pregnane X receptor
VDR: Vitamin D receptor
LXR: Liver X receptor
GR: Glucocorticoid receptor
S1PR2: Sphingosine-1-phosphate receptor 2 receptor
NMDA: N-methyl-D-aspartate
GABAA: γ-aminobutyric acid (GABA) type A (GABAA) receptor
TMN: Tuberomammillary nucleus
NSCs: Neural stem cells
SGZ: Subgranular zone
DG: Dentate gyrus
SVZ: Subventricular zone
FGF-15/19: Fibroblast growth factor 15/19
GLP-1: Glucagon-like peptide-1
DDP-4: Dipeptidyl peptidase IV
ROS: Reactive oxygen species
HE: Hepatic encephalopathy
AD: Alzheimer’s disease
Abeta: Beta-amyloid
PD: Parkinson’s disease (PD)
HD: Huntington’s disease
MS: Multiple sclerosis
CTX: Cerebrotendinous xanthomatosis
IL-1β: Interleukin-1 beta
NO: Nitric oxide
TNF-α: Tumor necrosis factorα
IFN-γ: Interferon-γ
IL-6: Interleukin-6
IL-10: Interleukin-10
MCP-1: Monocyte chemotactic protein-1
VCAM-1: Vascular cell adhesion 1
TGFβ: Transforming growth factorβ
SCI: Spinal cord injury
COX-2: Cyclooxygenase-2
iNOS: Inducible nitric oxide synthase
BMDMs: Bone marrow-derived macrophages
ERK: Extracellular signal-regulated kinase
JNK: c-Jun N-terminal kinase
MAPK: Mitogen-activated protein kinase
PI3K: Phosphatidylinositol 3-kinase
MR: Mineralcorticoid receptor (MR);
APP: Amyloid precursor protein
3-NP: 3-nitropropionic acid
SOD1: Superoxide dismutase-1
Nrf2: Nuclear factor erythroid 2 related factor 2
GPx: Glutathione peroxidase
HO-1: Heme oxygenase-1
MPTP: 1-methyl-4-phenyl-1,2,3,6-tetrahydropyridine
UPR: Unfolded protein response

## References

[1] Chiang JYL, Ferrell JM (2019). Bile acids as metabolic regulators and nutrient sensors. Annu Rev Nutr.

[2] Di Ciaula A, Garruti G, Lunardi Baccetto R, Molina-Molina E, Bonfrate L, Wang DQ, Portincasa P (2017). Bile Acid Physiology. Ann Hepatol.

[3] Russell DW (2003). The enzymes, regulation, and genetics of bile acid synthesis. Annu Rev Biochem.

[4] Takahashi S, Fukami T, Masuo Y, Brocker CN, Xie C, Krausz KW, Wolf CR, Henderson CJ, Gonzalez FJ (2016). Cyp2c70 is responsible for the species difference in bile acid metabolism between mice and humans. J Lipid Res.

[5] Hofmann AF, Hagey LR (2008). Bile acids: chemistry, pathochemistry, biology, pathobiology, and therapeutics. Cell Mol Life Sci.

[6] Eggert T, Bakonyi D, Hummel W (2014). Enzymatic routes for the synthesis of ursodeoxycholic acid. J Biotechnol.

[7] Tonin F, Otten LG, Arends I (2019). NAD(+) -Dependent Enzymatic Route for the epimerization of Hydroxysteroids. Chemsuschem.

[8] Wahlstrom A, Sayin SI, Marschall HU, Backhed F (2016). Intestinal crosstalk between bile acids and microbiota and its impact on host metabolism. Cell Metab.

[9] Dawson PA, Karpen SJ (2015). Intestinal transport and metabolism of bile acids. J Lipid Res.

[10] Hagenbuch B, Meier PJ (1994). Molecular cloning, chromosomal localization, and functional characterization of a human liver Na+/bile acid cotransporter. J Clin Invest.

[11] Jacquemin E, Hagenbuch B, Stieger B, Wolkoff AW, Meier PJ (1994). Expression cloning of a rat liver na(+)-independent organic anion transporter. Proc Natl Acad Sci U S A.

[12] Dawson PA, Hubbert M, Haywood J, Craddock AL, Zerangue N, Christian WV, Ballatori N (2005). The heteromeric organic solute transporter alpha-beta, Ostalpha-Ostbeta, is an ileal basolateral bile acid transporter. J Biol Chem.

[13] Schwarz M, Russell DW, Dietschy JM, Turley SD (1998). Marked reduction in bile acid synthesis in cholesterol 7alpha-hydroxylase-deficient mice does not lead to diminished tissue cholesterol turnover or to hypercholesterolemia. J Lipid Res.

[14] Rizzolo D, Buckley K, Kong B, Zhan L, Shen J, Stofan M, Brinker A, Goedken M, Buckley B, Guo GL (2019). Bile Acid Homeostasis in a cholesterol 7alpha-Hydroxylase and sterol 27-Hydroxylase double knockout mouse model. Hepatology.

[15] Rizzolo D, Kong B, Taylor RE, Brinker A, Goedken M, Buckley B, Guo GL (2021). Bile acid homeostasis in female mice deficient in Cyp7a1 and Cyp27a1. Acta Pharm Sin B.

[16] Bjorkhem I, Meaney S (2004). Brain cholesterol: long secret life behind a barrier. Arterioscler Thromb Vasc Biol.

[17] Zhang J, Liu Q (2015). Cholesterol metabolism and homeostasis in the brain. Protein Cell.

[18] Lund EG, Guileyardo JM, Russell DW (1999). cDNA cloning of cholesterol 24-hydroxylase, a mediator of cholesterol homeostasis in the brain. Proc Natl Acad Sci U S A.

[19] Bjorkhem I, Andersson U, Ellis E, Alvelius G, Ellegard L, Diczfalusy U, Sjovall J, Einarsson C (2001). From brain to bile. Evidence that conjugation and omega-hydroxylation are important for elimination of 24S-hydroxycholesterol (cerebrosterol) in humans. J Biol Chem.

[20] Li-Hawkins J, Lund EG, Bronson AD, Russell DW (2000). Expression cloning of an oxysterol 7alpha-hydroxylase selective for 24-hydroxycholesterol. J Biol Chem.

[21] Stiles AR, Kozlitina J, Thompson BM, McDonald JG, King KS, Russell DW (2014). Genetic, anatomic, and clinical determinants of human serum sterol and vitamin D levels. Proc Natl Acad Sci U S A.

[22] Bjorkhem I, Lutjohann D, Breuer O, Sakinis A, Wennmalm A (1997). Importance of a novel oxidative mechanism for elimination of brain cholesterol. Turnover of cholesterol and 24(S)-hydroxycholesterol in rat brain as measured with 18O2 techniques in vivo and in vitro. J Biol Chem.

[23] Kotti TJ, Ramirez DM, Pfeiffer BE, Huber KM, Russell DW (2006). Brain cholesterol turnover required for geranylgeraniol production and learning in mice. Proc Natl Acad Sci U S A.

[24] Lund EG, Xie C, Kotti T, Turley SD, Dietschy JM, Russell DW (2003). Knockout of the cholesterol 24-hydroxylase gene in mice reveals a brain-specific mechanism of cholesterol turnover. J Biol Chem.

[25] Boussicault L, Alves S, Lamaziere A, Planques A, Heck N, Moumne L, Despres G, Bolte S, Hu A, Pages C (2016). CYP46A1, the rate-limiting enzyme for cholesterol degradation, is neuroprotective in Huntington’s disease. Brain.

[26] Stiles AR, McDonald JG, Bauman DR, Russell DW (2009). CYP7B1: one cytochrome P450, two human genetic diseases, and multiple physiological functions. J Biol Chem.

[27] Wang M, Heo GY, Omarova S, Pikuleva IA, Turko IV (2012). Sample prefractionation for mass spectrometry quantification of low-abundance membrane proteins. Anal Chem.

[28] Ali Z, Heverin M, Olin M, Acimovic J, Lovgren-Sandblom A, Shafaati M, Bavner A, Meiner V, Leitersdorf E, Bjorkhem I (2013). On the regulatory role of side-chain hydroxylated oxysterols in the brain. Lessons from CYP27A1 transgenic and Cyp27a1(-/-) mice. J Lipid Res.

[29] Meaney S, Heverin M, Panzenboeck U, Ekstrom L, Axelsson M, Andersson U, Diczfalusy U, Pikuleva I, Wahren J, Sattler W (2007). Novel route for elimination of brain oxysterols across the blood-brain barrier: conversion into 7alpha-hydroxy-3-oxo-4-cholestenoic acid. J Lipid Res.

[30] Repa JJ, Lund EG, Horton JD, Leitersdorf E, Russell DW, Dietschy JM, Turley SD (2000). Disruption of the sterol 27-hydroxylase gene in mice results in hepatomegaly and hypertriglyceridemia. Reversal by cholic acid feeding. J Biol Chem.

[31] Bavner A, Shafaati M, Hansson M, Olin M, Shpitzen S, Meiner V, Leitersdorf E, Bjorkhem I (2010). On the mechanism of accumulation of cholestanol in the brain of mice with a disruption of sterol 27-hydroxylase. J Lipid Res.

[32] Wu Z, Martin KO, Javitt NB, Chiang JY (1999). Structure and functions of human oxysterol 7alpha-hydroxylase cDNAs and gene CYP7B1. J Lipid Res.

[33] Rose KA, Stapleton G, Dott K, Kieny MP, Best R, Schwarz M, Russell DW, Bjorkhem I, Seckl J, Lathe R (1997). Cyp7b, a novel brain cytochrome P450, catalyzes the synthesis of neurosteroids 7alpha-hydroxy dehydroepiandrosterone and 7alpha-hydroxy pregnenolone. Proc Natl Acad Sci U S A.

[34] Ridlon JM, Kang DJ, Hylemon PB (2006). Bile salt biotransformations by human intestinal bacteria. J Lipid Res.

[35] Ridlon JM, Harris SC, Bhowmik S, Kang DJ, Hylemon PB (2016). Consequences of bile salt biotransformations by intestinal bacteria. Gut Microbes.

[36] Molinero N, Ruiz L, Sanchez B, Margolles A, Delgado S (2019). Intestinal Bacteria interplay with bile and cholesterol metabolism: implications on host physiology. Front Physiol.

[37] Sayin SI, Wahlstrom A, Felin J, Jantti S, Marschall HU, Bamberg K, Angelin B, Hyotylainen T, Oresic M, Backhed F (2013). Gut microbiota regulates bile acid metabolism by reducing the levels of tauro-beta-muricholic acid, a naturally occurring FXR antagonist. Cell Metab.

[38] Joyce SA, MacSharry J, Casey PG, Kinsella M, Murphy EF, Shanahan F, Hill C, Gahan CG (2014). Regulation of host weight gain and lipid metabolism by bacterial bile acid modification in the gut. Proc Natl Acad Sci U S A.

[39] Morais LH, Schreiber HLt, Mazmanian SK (2021). The gut microbiota-brain axis in behaviour and brain disorders. Nat Rev Microbiol.

[40] Park J, Kim CH (2021). Regulation of common neurological disorders by gut microbial metabolites. Exp Mol Med.

[41] Mano N, Goto T, Uchida M, Nishimura K, Ando M, Kobayashi N, Goto J (2004). Presence of protein-bound unconjugated bile acids in the cytoplasmic fraction of rat brain. J Lipid Res.

[42] Baloni P, Funk CC, Yan J, Yurkovich JT, Kueider-Paisley A, Nho K, Heinken A, Jia W, Mahmoudiandehkordi S, Louie G (2020). Metabolic network analysis reveals altered bile acid synthesis and metabolism in Alzheimer’s Disease. Cell Rep Med.

[43] Zheng X, Chen T, Zhao A, Wang X, Xie G, Huang F, Liu J, Zhao Q, Wang S, Wang C (2016). The Brain Metabolome of male rats across the Lifespan. Sci Rep.

[44] Pan X, Elliott CT, McGuinness B, Passmore P, Kehoe PG, Holscher C, McClean PL, Graham SF, Green BD. Metabolomic profiling of bile acids in clinical and experimental samples of Alzheimer’s Disease. Metabolites 2017, 7(2). 10.3390/metabo7020028 PMC5487999 28629125

[45] Roda A, Minutello A, Angellotti MA, Fini A (1990). Bile acid structure-activity relationship: evaluation of bile acid lipophilicity using 1-octanol/water partition coefficient and reverse phase HPLC. J Lipid Res.

[46] Kitazawa T, Terasaki T, Suzuki H, Kakee A, Sugiyama Y (1998). Efflux of taurocholic acid across the blood-brain barrier: interaction with cyclic peptides. J Pharmacol Exp Ther.

[47] Palmela I, Correia L, Silva RF, Sasaki H, Kim KS, Brites D, Brito MA (2015). Hydrophilic bile acids protect human blood-brain barrier endothelial cells from disruption by unconjugated bilirubin: an in vitro study. Front Neurosci.

[48] Higashi T, Watanabe S, Tomaru K, Yamazaki W, Yoshizawa K, Ogawa S, Nagao H, Minato K, Maekawa M, Mano N (2017). Unconjugated bile acids in rat brain: Analytical method based on LC/ESI-MS/MS with chemical derivatization and estimation of their origin by comparison to serum levels. Steroids.

[49] Choudhuri S, Cherrington NJ, Li N, Klaassen CD (2003). Constitutive expression of various xenobiotic and endobiotic transporter mRNAs in the choroid plexus of rats. Drug Metab Dispos.

[50] Ose A, Kusuhara H, Endo C, Tohyama K, Miyajima M, Kitamura S, Sugiyama Y (2010). Functional characterization of mouse organic anion transporting peptide 1a4 in the uptake and efflux of drugs across the blood-brain barrier. Drug Metab Dispos.

[51] McMillin M, Frampton G, Quinn M, Divan A, Grant S, Patel N, Newell-Rogers K, DeMorrow S (2015). Suppression of the HPA Axis during Cholestasis can be attributed to hypothalamic bile Acid Signaling. Mol Endocrinol.

[52] Nizamutdinov D, DeMorrow S, McMillin M, Kain J, Mukherjee S, Zeitouni S, Frampton G, Bricker PC, Hurst J, Shapiro LA (2017). Hepatic alterations are accompanied by changes to bile acid transporter-expressing neurons in the hypothalamus after traumatic brain injury. Sci Rep.

[53] Perino A, Velazquez-Villegas LA, Bresciani N, Sun Y, Huang Q, Fenelon VS, Castellanos-Jankiewicz A, Zizzari P, Bruschetta G, Jin S (2021). Central anorexigenic actions of bile acids are mediated by TGR5. Nat Metab.

[54] Greenwood J, Adu J, Davey AJ, Abbott NJ, Bradbury MW (1991). The effect of bile salts on the permeability and ultrastructure of the perfused, energy-depleted, rat blood-brain barrier. J Cereb Blood Flow Metab.

[55] Quinn M, McMillin M, Galindo C, Frampton G, Pae HY, DeMorrow S (2014). Bile acids permeabilize the blood brain barrier after bile duct ligation in rats via Rac1-dependent mechanisms. Dig Liver Dis.

[56] Grant SM, DeMorrow S. Bile Acid Signaling in neurodegenerative and neurological Disorders. Int J Mol Sci 2020, 21(17). 10.3390/ijms21175982 PMC7503576 32825239

[57] Schaap FG, Trauner M, Jansen PL (2014). Bile acid receptors as targets for drug development. Nat Rev Gastroenterol Hepatol.

[58] Huang C, Wang J, Hu W, Wang C, Lu X, Tong L, Wu F, Zhang W (2016). Identification of functional farnesoid X receptors in brain neurons. FEBS Lett.

[59] McMillin M, Frampton G, Quinn M, Ashfaq S, de los Santos M 3rd, Grant S, DeMorrow S. Bile Acid Signaling is involved in the neurological decline in a murine model of Acute Liver failure. Am J Pathol. 2016;186(2):312–23. 10.1016/j.ajpath.2015.10.005 PMC4729266 26683664

[60] Huang F, Wang T, Lan Y, Yang L, Pan W, Zhu Y, Lv B, Wei Y, Shi H, Wu H (2015). Deletion of mouse FXR gene disturbs multiple neurotransmitter systems and alters neurobehavior. Front Behav Neurosci.

[61] Montes de Oca Balderas P. Flux-independent NMDAR Signaling: Molecular Mediators, Cellular Functions, and complexities. Int J Mol Sci 2018, 19(12). 10.3390/ijms19123800 PMC6321296 30501045

[62] Cicek SS. Structure-dependent activity of natural GABA(A) receptor modulators. Molecules 2018, 23(7). 10.3390/molecules23071512 PMC6100244 29932138

[63] Hosie AM, Wilkins ME, da Silva HM, Smart TG (2006). Endogenous neurosteroids regulate GABAA receptors through two discrete transmembrane sites. Nature.

[64] Liao WL, Heo GY, Dodder NG, Reem RE, Mast N, Huang S, Dipatre PL, Turko IV, Pikuleva IA (2011). Quantification of cholesterol-metabolizing P450s CYP27A1 and CYP46A1 in neural tissues reveals a lack of enzyme-product correlations in human retina but not human brain. J Proteome Res.

[65] Schubring SR, Fleischer W, Lin JS, Haas HL, Sergeeva OA (2012). The bile steroid chenodeoxycholate is a potent antagonist at NMDA and GABA(A) receptors. Neurosci Lett.

[66] Fujita A, Bonnavion P, Wilson MH, Mickelsen LE, Bloit J, de Lecea L, Jackson AC (2017). Hypothalamic tuberomammillary nucleus neurons: electrophysiological diversity and essential role in Arousal Stability. J Neurosci.

[67] Xie JF, Fan K, Wang C, Xie P, Hou M, Xin L, Cui GF, Wang LX, Shao YF, Hou YP (2017). Inactivation of the Tuberomammillary Nucleus by GABA(A) receptor agonist promotes slow Wave Sleep in freely moving rats and histamine-treated rats. Neurochem Res.

[68] Yanovsky Y, Schubring SR, Yao Q, Zhao Y, Li S, May A, Haas HL, Lin JS, Sergeeva OA (2012). Waking action of ursodeoxycholic acid (UDCA) involves histamine and GABAA receptor block. PLoS ONE.

[69] Xavier JM, Morgado AL, Rodrigues CM, Sola S (2014). Tauroursodeoxycholic acid increases neural stem cell pool and neuronal conversion by regulating mitochondria-cell cycle retrograde signaling. Cell Cycle.

[70] Soares R, Ribeiro FF, Xapelli S, Genebra T, Ribeiro MF, Sebastiao AM, Rodrigues CMP, Sola S (2018). Tauroursodeoxycholic acid enhances mitochondrial Biogenesis, neural stem Cell Pool, and early neurogenesis in adult rats. Mol Neurobiol.

[71] Mertens KL, Kalsbeek A, Soeters MR, Eggink HM (2017). Bile Acid Signaling Pathways from the enterohepatic circulation to the Central Nervous System. Front Neurosci.

[72] Kliewer SA, Mangelsdorf DJ (2015). Bile acids as hormones: the FXR-FGF15/19 pathway. Dig Dis.

[73] Gadaleta RM, Moschetta A (2019). Metabolic messengers: fibroblast growth factor 15/19. Nat Metab.

[74] Hsuchou H, Pan W, Kastin AJ (2013). Fibroblast growth factor 19 entry into brain. Fluids Barriers CNS.

[75] Ryan KK, Kohli R, Gutierrez-Aguilar R, Gaitonde SG, Woods SC, Seeley RJ (2013). Fibroblast growth factor-19 action in the brain reduces food intake and body weight and improves glucose tolerance in male rats. Endocrinology.

[76] Miyake A, Itoh N (1996). Rat fibroblast growth factor receptor-4 mRNA in the brain is preferentially expressed in cholinergic neurons in the medial habenular nucleus. Neurosci Lett.

[77] Marcelin G, Jo YH, Li X, Schwartz GJ, Zhang Y, Dun NJ, Lyu RM, Blouet C, Chang JK, Chua S (2014). Jr.: central action of FGF19 reduces hypothalamic AGRP/NPY neuron activity and improves glucose metabolism. Mol Metab.

[78] Liu S, Marcelin G, Blouet C, Jeong JH, Jo YH, Schwartz GJ, Chua S (2018). A gut-brain axis regulating glucose metabolism mediated by bile acids and competitive fibroblast growth factor actions at the hypothalamus. Mol Metab.

[79] Brighton CA, Rievaj J, Kuhre RE, Glass LL, Schoonjans K, Holst JJ, Gribble FM, Reimann F (2015). Bile acids trigger GLP-1 release predominantly by accessing basolaterally located G protein-coupled bile acid receptors. Endocrinology.

[80] Katsuma S, Hirasawa A, Tsujimoto G (2005). Bile acids promote glucagon-like peptide-1 secretion through TGR5 in a murine enteroendocrine cell line STC-1. Biochem Biophys Res Commun.

[81] Ullmer C, Alvarez Sanchez R, Sprecher U, Raab S, Mattei P, Dehmlow H, Sewing S, Iglesias A, Beauchamp J, Conde-Knape K (2013). Systemic bile acid sensing by G protein-coupled bile acid receptor 1 (GPBAR1) promotes PYY and GLP-1 release. Br J Pharmacol.

[82] Yamamoto H, Kishi T, Lee CE, Choi BJ, Fang H, Hollenberg AN, Drucker DJ, Elmquist JK (2003). Glucagon-like peptide-1-responsive catecholamine neurons in the area postrema link peripheral glucagon-like peptide-1 with central autonomic control sites. J Neurosci.

[83] Orskov C, Poulsen SS, Moller M, Holst JJ (1996). Glucagon-like peptide I receptors in the subfornical organ and the area postrema are accessible to circulating glucagon-like peptide I. Diabetes.

[84] Abbott CR, Monteiro M, Small CJ, Sajedi A, Smith KL, Parkinson JR, Ghatei MA, Bloom SR (2005). The inhibitory effects of peripheral administration of peptide YY(3–36) and glucagon-like peptide-1 on food intake are attenuated by ablation of the vagal-brainstem-hypothalamic pathway. Brain Res.

[85] Ruttimann EB, Arnold M, Hillebrand JJ, Geary N, Langhans W (2009). Intrameal hepatic portal and intraperitoneal infusions of glucagon-like peptide-1 reduce spontaneous meal size in the rat via different mechanisms. Endocrinology.

[86] Cork SC, Richards JE, Holt MK, Gribble FM, Reimann F, Trapp S (2015). Distribution and characterisation of glucagon-like peptide-1 receptor expressing cells in the mouse brain. Mol Metab.

[87] Richards P, Parker HE, Adriaenssens AE, Hodgson JM, Cork SC, Trapp S, Gribble FM, Reimann F (2014). Identification and characterization of GLP-1 receptor-expressing cells using a new transgenic mouse model. Diabetes.

[88] Kastin AJ, Akerstrom V, Pan W (2002). Interactions of glucagon-like peptide-1 (GLP-1) with the blood-brain barrier. J Mol Neurosci.

[89] Borgmann D, Ciglieri E, Biglari N, Brandt C, Cremer AL, Backes H, Tittgemeyer M, Wunderlich FT, Bruning JC, Fenselau H (2021). Gut-brain communication by distinct sensory neurons differently controls feeding and glucose metabolism. Cell Metab.

[90] Drucker DJ, Nauck MA (2006). The incretin system: glucagon-like peptide-1 receptor agonists and dipeptidyl peptidase-4 inhibitors in type 2 diabetes. Lancet.

[91] Punjabi M, Arnold M, Ruttimann E, Graber M, Geary N, Pacheco-Lopez G, Langhans W (2014). Circulating glucagon-like peptide-1 (GLP-1) inhibits eating in male rats by acting in the hindbrain and without inducing avoidance. Endocrinology.

[92] Hayes MR, Skibicka KP, Grill HJ (2008). Caudal brainstem processing is sufficient for behavioral, sympathetic, and parasympathetic responses driven by peripheral and hindbrain glucagon-like-peptide-1 receptor stimulation. Endocrinology.

[93] Williams DL, Baskin DG, Schwartz MW (2009). Evidence that intestinal glucagon-like peptide-1 plays a physiological role in satiety. Endocrinology.

[94] Holst JJ (2007). The physiology of glucagon-like peptide 1. Physiol Rev.

[95] Perez MJ, Briz O (2009). Bile-acid-induced cell injury and protection. World J Gastroenterol.

[96] Rose CF, Amodio P, Bajaj JS, Dhiman RK, Montagnese S, Taylor-Robinson SD, Vilstrup H, Jalan R (2020). Hepatic encephalopathy: novel insights into classification, pathophysiology and therapy. J Hepatol.

[97] Xie G, Wang X, Jiang R, Zhao A, Yan J, Zheng X, Huang F, Liu X, Panee J, Rajani C (2018). Dysregulated bile acid signaling contributes to the neurological impairment in murine models of acute and chronic liver failure. EBioMedicine.

[98] Weiss N, Barbier Saint Hilaire P, Colsch B, Isnard F, Attala S, Schaefer A, Amador MD, Rudler M, Lamari F, Sedel F (2016). Cerebrospinal fluid metabolomics highlights dysregulation of energy metabolism in overt hepatic encephalopathy. J Hepatol.

[99] Tripodi V, Contin M, Fernandez MA, Lemberg A (2012). Bile acids content in brain of common duct ligated rats. Ann Hepatol.

[100] McMillin M, Frampton G, Grant S, Khan S, Diocares J, Petrescu A, Wyatt A, Kain J, Jefferson B, DeMorrow S (2017). Bile acid-mediated sphingosine-1-Phosphate receptor 2 signaling promotes neuroinflammation during hepatic encephalopathy in mice. Front Cell Neurosci.

[101] Lane CA, Hardy J, Schott JM (2018). Alzheimer’s disease. Eur J Neurol.

[102] Olazaran J, Gil-de-Gomez L, Rodriguez-Martin A, Valenti-Soler M, Frades-Payo B, Marin-Munoz J, Antunez C, Frank-Garcia A, Acedo-Jimenez C, Morlan-Gracia L (2015). A blood-based, 7-metabolite signature for the early diagnosis of Alzheimer’s disease. J Alzheimers Dis.

[103] Mapstone M, Cheema AK, Fiandaca MS, Zhong X, Mhyre TR, MacArthur LH, Hall WJ, Fisher SG, Peterson DR, Haley JM (2014). Plasma phospholipids identify antecedent memory impairment in older adults. Nat Med.

[104] Marksteiner J, Blasko I, Kemmler G, Koal T, Humpel C (2018). Bile acid quantification of 20 plasma metabolites identifies lithocholic acid as a putative biomarker in Alzheimer’s disease. Metabolomics.

[105] MahmoudianDehkordi S, Arnold M, Nho K, Ahmad S, Jia W, Xie G, Louie G, Kueider-Paisley A, Moseley MA, Thompson JW (2019). Altered bile acid profile associates with cognitive impairment in Alzheimer’s disease-An emerging role for gut microbiome. Alzheimers Dement.

[106] Nho K, Kueider-Paisley A, MahmoudianDehkordi S, Arnold M, Risacher SL, Louie G, Blach C, Baillie R, Han X, Kastenmuller G (2019). Altered bile acid profile in mild cognitive impairment and Alzheimer’s disease: relationship to neuroimaging and CSF biomarkers. Alzheimers Dement.

[107] Shao Y, Li T, Liu Z, Wang X, Xu X, Li S, Xu G, Le W (2021). Comprehensive metabolic profiling of Parkinson’s disease by liquid chromatography-mass spectrometry. Mol Neurodegener.

[108] Li P, Killinger BA, Ensink E, Beddows I, Yilmaz A, Lubben N, Lamp J, Schilthuis M, Vega IE, Woltjer R et al. Gut microbiota dysbiosis is Associated with elevated bile acids in Parkinson’s Disease. Metabolites 2021, 11(1). 10.3390/metabo11010029 PMC7823437 33406628

[109] Yilmaz A, Ugur Z, Ustun I, Akyol S, Bahado-Singh RO, Maddens M, Aasly JO, Graham SF. Metabolic profiling of CSF from people suffering from sporadic and LRRK2 Parkinson’s Disease: a pilot study. Cells 2020, 9(11). 10.3390/cells9112394 PMC7693941 33142859

[110] Graham SF, Rey NL, Ugur Z, Yilmaz A, Sherman E, Maddens M, Bahado-Singh RO, Becker K, Schulz E, Meyerdirk LK et al. Metabolomic profiling of bile acids in an experimental model of Prodromal Parkinson’s Disease. Metabolites 2018, 8(4). 10.3390/metabo8040071 PMC6316593 30384419

[111] McColgan P, Tabrizi SJ (2018). Huntington’s disease: a clinical review. Eur J Neurol.

[112] Kacher R, Mounier C, Caboche J, Betuing S (2022). Altered cholesterol homeostasis in Huntington’s Disease. Front Aging Neurosci.

[113] Leoni V, Mariotti C, Tabrizi SJ, Valenza M, Wild EJ, Henley SM, Hobbs NZ, Mandelli ML, Grisoli M, Bjorkhem I (2008). Plasma 24S-hydroxycholesterol and caudate MRI in pre-manifest and early Huntington’s disease. Brain.

[114] Leoni V, Long JD, Mills JA, Di Donato S, Paulsen JS (2013). Group P-Hs: plasma 24S-hydroxycholesterol correlation with markers of Huntington disease progression. Neurobiol Dis.

[115] Valenza M, Leoni V, Karasinska JM, Petricca L, Fan J, Carroll J, Pouladi MA, Fossale E, Nguyen HP, Riess O (2010). Cholesterol defect is marked across multiple rodent models of Huntington’s disease and is manifest in astrocytes. J Neurosci.

[116] Verrips A, van Engelen BG, Wevers RA, van Geel BM, Cruysberg JR, van den Heuvel LP, Keyser A, Gabreels FJ (2000). Presence of diarrhea and absence of tendon xanthomas in patients with cerebrotendinous xanthomatosis. Arch Neurol.

[117] Verrips A, Hoefsloot LH, Steenbergen GC, Theelen JP, Wevers RA, Gabreels FJ, van Engelen BG, van den Heuvel LP (2000). Clinical and molecular genetic characteristics of patients with cerebrotendinous xanthomatosis. Brain.

[118] Wong JC, Walsh K, Hayden D, Eichler FS (2018). Natural history of neurological abnormalities in cerebrotendinous xanthomatosis. J Inherit Metab Dis.

[119] Nie S, Chen G, Cao X, Zhang Y (2014). Cerebrotendinous xanthomatosis: a comprehensive review of pathogenesis, clinical manifestations, diagnosis, and management. Orphanet J Rare Dis.

[120] Mandia D, Chaussenot A, Besson G, Lamari F, Castelnovo G, Curot J, Duval F, Giral P, Lecerf JM, Roland D (2019). Cholic acid as a treatment for cerebrotendinous xanthomatosis in adults. J Neurol.

[121] Koyama S, Sekijima Y, Ogura M, Hori M, Matsuki K, Miida T, Harada-Shiba M (2021). Cerebrotendinous Xanthomatosis: Molecular Pathogenesis, clinical spectrum, diagnosis, and Disease-Modifying treatments. J Atheroscler Thromb.

[122] Stelten BML, Lycklama ANGJ, Hendriks E, Kluijtmans LAJ, Wevers RA, Verrips A (2022). Long-term MRI findings in patients with Cerebrotendinous Xanthomatosis treated with Chenodeoxycholic Acid. Neurology.

[123] Amador MDM, Masingue M, Debs R, Lamari F, Perlbarg V, Roze E, Degos B, Mochel F (2018). Treatment with chenodeoxycholic acid in cerebrotendinous xanthomatosis: clinical, neurophysiological, and quantitative brain structural outcomes. J Inherit Metab Dis.

[124] Khalaf K, Tornese P, Cocco A, Albanese A (2022). Tauroursodeoxycholic acid: a potential therapeutic tool in neurodegenerative diseases. Transl Neurodegener.

[125] Wolf SA, Boddeke HW, Kettenmann H (2017). Microglia in Physiology and Disease. Annu Rev Physiol.

[126] Matias I, Morgado J, Gomes FCA (2019). Astrocyte heterogeneity: impact to Brain Aging and Disease. Front Aging Neurosci.

[127] Stephenson J, Nutma E, van der Valk P, Amor S (2018). Inflammation in CNS neurodegenerative diseases. Immunology.

[128] Joo SS, Kang HC, Won TJ, Lee DI (2003). Ursodeoxycholic acid inhibits pro-inflammatory repertoires, IL-1 beta and nitric oxide in rat microglia. Arch Pharm Res.

[129] Joo SS, Won TJ, Lee DI (2004). Potential role of ursodeoxycholic acid in suppression of nuclear factor kappa B in microglial cell line (BV-2). Arch Pharm Res.

[130] Jiang C, Shen D, Li K, Wang H, Sang W, Qi H (2022). Protective Effects of Ursodeoxycholic Acid against oxidative stress and Neuroinflammation through Mitogen-Activated protein kinases pathway in MPTP-Induced Parkinson Disease. Clin Neuropharmacol.

[131] Yanguas-Casas N, Barreda-Manso MA, Nieto-Sampedro M, Romero-Ramirez L (2014). Tauroursodeoxycholic acid reduces glial cell activation in an animal model of acute neuroinflammation. J Neuroinflammation.

[132] Yanguas-Casas N, Barreda-Manso MA, Perez-Rial S, Nieto-Sampedro M, Romero-Ramirez L (2017). TGFbeta contributes to the anti-inflammatory Effects of Tauroursodeoxycholic Acid on an animal model of Acute Neuroinflammation. Mol Neurobiol.

[133] Yanguas-Casas N, Barreda-Manso MA, Nieto-Sampedro M, Romero-Ramirez L (2017). TUDCA: an agonist of the bile acid receptor GPBAR1/TGR5 with anti-inflammatory Effects in Microglial cells. J Cell Physiol.

[134] Wu X, Liu C, Chen L, Du YF, Hu M, Reed MN, Long Y, Suppiramaniam V, Hong H, Tang SS (2019). Protective effects of tauroursodeoxycholic acid on lipopolysaccharide-induced cognitive impairment and neurotoxicity in mice. Int Immunopharmacol.

[135] Sweis R, Biller J (2017). Systemic complications of spinal cord Injury. Curr Neurol Neurosci Rep.

[136] Kim SJ, Ko WK, Jo MJ, Arai Y, Choi H, Kumar H, Han IB, Sohn S (2018). Anti-inflammatory effect of tauroursodeoxycholic acid in RAW 264.7 macrophages, bone marrow-derived macrophages, BV2 microglial cells, and spinal cord injury. Sci Rep.

[137] Hou Y, Luan J, Huang T, Deng T, Li X, Xiao Z, Zhan J, Luo D, Hou Y, Xu L (2021). Tauroursodeoxycholic acid alleviates secondary injury in spinal cord injury mice by reducing oxidative stress, apoptosis, and inflammatory response. J Neuroinflammation.

[138] Romero-Ramirez L, Garcia-Rama C, Wu S, Mey J (2022). Bile acids attenuate PKM2 pathway activation in proinflammatory microglia. Sci Rep.

[139] Han GH, Kim SJ, Ko WK, Lee D, Lee JS, Nah H, Han IB, Sohn S (2020). Injectable Hydrogel containing tauroursodeoxycholic acid for anti-neuroinflammatory Therapy after spinal cord Injury in rats. Mol Neurobiol.

[140] Han GH, Kim SJ, Ko WK, Lee D, Han IB, Sheen SH, Hong JB, Sohn S (2021). Transplantation of tauroursodeoxycholic acid-inducing M2-phenotype macrophages promotes an anti-neuroinflammatory effect and functional recovery after spinal cord injury in rats. Cell Prolif.

[141] Wu S, Garcia-Rama C, Romero-Ramirez L, de Munter J, Wolters EC, Kramer BW, Mey J. Tauroursodeoxycholic acid reduces Neuroinflammation but does not support long term functional recovery of rats with spinal cord Injury. Biomedicines 2022, 10(7). 10.3390/biomedicines10071501 PMC9313003 35884805

[142] Nunes AF, Amaral JD, Lo AC, Fonseca MB, Viana RJ, Callaerts-Vegh Z, D’Hooge R, Rodrigues CM (2012). TUDCA, a bile acid, attenuates amyloid precursor protein processing and amyloid-beta deposition in APP/PS1 mice. Mol Neurobiol.

[143] Dionisio PA, Amaral JD, Ribeiro MF, Lo AC, D’Hooge R, Rodrigues CM (2015). Amyloid-beta pathology is attenuated by tauroursodeoxycholic acid treatment in APP/PS1 mice after disease onset. Neurobiol Aging.

[144] Cuevas E, Burks S, Raymick J, Robinson B, Gomez-Crisostomo NP, Escudero-Lourdes C, Lopez AGG, Chigurupati S, Hanig J, Ferguson SA (2022). Tauroursodeoxycholic acid (TUDCA) is neuroprotective in a chronic mouse model of Parkinson’s disease. Nutr Neurosci.

[145] Mendes MO, Rosa AI, Carvalho AN, Nunes MJ, Dionisio P, Rodrigues E, Costa D, Duarte-Silva S, Maciel P, Rodrigues CMP (2019). Neurotoxic effects of MPTP on mouse cerebral cortex: modulation of neuroinflammation as a neuroprotective strategy. Mol Cell Neurosci.

[146] Reich DS, Lucchinetti CF, Calabresi PA (2018). Multiple sclerosis. N Engl J Med.

[147] Bhargava P, Smith MD, Mische L, Harrington E, Fitzgerald KC, Martin K, Kim S, Reyes AA, Gonzalez-Cardona J, Volsko C (2020). Bile acid metabolism is altered in multiple sclerosis and supplementation ameliorates neuroinflammation. J Clin Invest.

[148] Noailles A, Fernandez-Sanchez L, Lax P, Cuenca N (2014). Microglia activation in a model of retinal degeneration and TUDCA neuroprotective effects. J Neuroinflammation.

[149] Murase H, Tsuruma K, Shimazawa M, Hara H (2015). TUDCA promotes phagocytosis by Retinal Pigment Epithelium via MerTK activation. Invest Ophthalmol Vis Sci.

[150] Radi E, Formichi P, Battisti C, Federico A (2014). Apoptosis and oxidative stress in neurodegenerative diseases. J Alzheimers Dis.

[151] Rodrigues CM, Sola S, Silva R, Brites D (2000). Bilirubin and amyloid-beta peptide induce cytochrome c release through mitochondrial membrane permeabilization. Mol Med.

[152] Ramalho RM, Ribeiro PS, Sola S, Castro RE, Steer CJ, Rodrigues CM (2004). Inhibition of the E2F-1/p53/Bax pathway by tauroursodeoxycholic acid in amyloid beta-peptide-induced apoptosis of PC12 cells. J Neurochem.

[153] Viana RJ, Ramalho RM, Nunes AF, Steer CJ, Rodrigues CM (2010). Modulation of amyloid-beta peptide-induced toxicity through inhibition of JNK nuclear localization and caspase-2 activation. J Alzheimers Dis.

[154] Ramalho RM, Borralho PM, Castro RE, Sola S, Steer CJ, Rodrigues CM (2006). Tauroursodeoxycholic acid modulates p53-mediated apoptosis in Alzheimer’s disease mutant neuroblastoma cells. J Neurochem.

[155] Rodrigues CM, Sola S, Brito MA, Brondino CD, Brites D, Moura JJ (2001). Amyloid beta-peptide disrupts mitochondrial membrane lipid and protein structure: protective role of tauroursodeoxycholate. Biochem Biophys Res Commun.

[156] Sola S, Castro RE, Laires PA, Steer CJ, Rodrigues CM (2003). Tauroursodeoxycholic acid prevents amyloid-beta peptide-induced neuronal death via a phosphatidylinositol 3-kinase-dependent signaling pathway. Mol Med.

[157] Sola S, Amaral JD, Borralho PM, Ramalho RM, Castro RE, Aranha MM, Steer CJ, Rodrigues CM (2006). Functional modulation of nuclear steroid receptors by tauroursodeoxycholic acid reduces amyloid beta-peptide-induced apoptosis. Mol Endocrinol.

[158] Ramalho RM, Viana RJ, Low WC, Steer CJ, Rodrigues CM (2008). Bile acids and apoptosis modulation: an emerging role in experimental Alzheimer’s disease. Trends Mol Med.

[159] Lo AC, Callaerts-Vegh Z, Nunes AF, Rodrigues CM, D’Hooge R (2013). Tauroursodeoxycholic acid (TUDCA) supplementation prevents cognitive impairment and amyloid deposition in APP/PS1 mice. Neurobiol Dis.

[160] Duan WM, Rodrigues CM, Zhao LR, Steer CJ, Low WC (2002). Tauroursodeoxycholic acid improves the survival and function of nigral transplants in a rat model of Parkinson’s disease. Cell Transpl.

[161] Keene CD, Rodrigues CM, Eich T, Linehan-Stieers C, Abt A, Kren BT, Steer CJ, Low WC (2001). A bile acid protects against motor and cognitive deficits and reduces striatal degeneration in the 3-nitropropionic acid model of Huntington’s disease. Exp Neurol.

[162] Keene CD, Rodrigues CM, Eich T, Chhabra MS, Steer CJ, Low WC (2002). Tauroursodeoxycholic acid, a bile acid, is neuroprotective in a transgenic animal model of Huntington’s disease. Proc Natl Acad Sci U S A.

[163] Dong Y, Miao L, Hei L, Lin L, Ding H (2015). Neuroprotective effects and impact on caspase-12 expression of tauroursodeoxycholic acid after acute spinal cord injury in rats. Int J Clin Exp Pathol.

[164] Colak A, Kelten B, Sagmanligil A, Akdemir O, Karaoglan A, Sahan E, Celik O, Barut S (2008). Tauroursodeoxycholic acid and secondary damage after spinal cord injury in rats. J Clin Neurosci.

[165] Miao L, Dong Y, Zhou FB, Chang YL, Suo ZG, Ding HQ (2018). Protective effect of tauroursodeoxycholic acid on the autophagy of nerve cells in rats with acute spinal cord injury. Eur Rev Med Pharmacol Sci.

[166] Dong Y, Yang S, Fu B, Liu F, Zhou S, Ding H, Ma W (2020). Mechanism of tauroursodeoxycholic acid-mediated neuronal protection after acute spinal cord injury through AKT signaling pathway in rats. Int J Clin Exp Pathol.

[167] Zhang Z, Chen J, Chen F, Yu D, Li R, Lv C, Wang H, Li H, Li J, Cai Y (2018). Tauroursodeoxycholic acid alleviates secondary injury in the spinal cord via up-regulation of CIBZ gene. Cell Stress Chaperones.

[168] Chang Y, Yang T, Ding H, Wang Z, Liang Q (2022). Tauroursodeoxycholic acid protects rat spinal cord neurons after mechanical injury through regulating neuronal autophagy. Neurosci Lett.

[169] Hulisz D (2018). Amyotrophic lateral sclerosis: disease state overview. Am J Manag Care.

[170] Parry GJ, Rodrigues CM, Aranha MM, Hilbert SJ, Davey C, Kelkar P, Low WC, Steer CJ (2010). Safety, tolerability, and cerebrospinal fluid penetration of ursodeoxycholic acid in patients with amyotrophic lateral sclerosis. Clin Neuropharmacol.

[171] Min JH, Hong YH, Sung JJ, Kim SM, Lee JB, Lee KW (2012). Oral solubilized ursodeoxycholic acid therapy in amyotrophic lateral sclerosis: a randomized cross-over trial. J Korean Med Sci.

[172] Elia AE, Lalli S, Monsurro MR, Sagnelli A, Taiello AC, Reggiori B, La Bella V, Tedeschi G, Albanese A (2016). Tauroursodeoxycholic acid in the treatment of patients with amyotrophic lateral sclerosis. Eur J Neurol.

[173] Thams S, Lowry ER, Larraufie MH, Spiller KJ, Li H, Williams DJ, Hoang P, Jiang E, Williams LA, Sandoe J (2019). A stem cell-based screening platform identifies compounds that desensitize motor neurons to endoplasmic reticulum stress. Mol Ther.

[174] Vaz AR, Cunha C, Gomes C, Schmucki N, Barbosa M, Brites D (2015). Glycoursodeoxycholic acid reduces matrix metalloproteinase-9 and caspase-9 activation in a cellular model of superoxide dismutase-1 neurodegeneration. Mol Neurobiol.

[175] Rosa AI, Duarte-Silva S, Silva-Fernandes A, Nunes MJ, Carvalho AN, Rodrigues E, Gama MJ, Rodrigues CMP, Maciel P, Castro-Caldas M (2018). Tauroursodeoxycholic acid improves motor symptoms in a mouse model of Parkinson’s Disease. Mol Neurobiol.

[176] Rosa AI, Fonseca I, Nunes MJ, Moreira S, Rodrigues E, Carvalho AN, Rodrigues CMP, Gama MJ, Castro-Caldas M (2017). Novel insights into the antioxidant role of tauroursodeoxycholic acid in experimental models of Parkinson’s disease. Biochim Biophys Acta Mol Basis Dis.

[177] Moreira S, Fonseca I, Nunes MJ, Rosa A, Lemos L, Rodrigues E, Carvalho AN, Outeiro TF, Rodrigues CMP, Gama MJ (2017). Nrf2 activation by tauroursodeoxycholic acid in experimental models of Parkinson’s disease. Exp Neurol.

[178] Castro-Caldas M, Carvalho AN, Rodrigues E, Henderson CJ, Wolf CR, Rodrigues CM, Gama MJ (2012). Tauroursodeoxycholic acid prevents MPTP-induced dopaminergic cell death in a mouse model of Parkinson’s disease. Mol Neurobiol.

[179] Kusaczuk M. Tauroursodeoxycholate-bile acid with chaperoning activity: Molecular and Cellular Effects and therapeutic perspectives. Cells 2019, 8(12). 10.3390/cells8121471 PMC6952947 31757001

[180] van der Harg JM, Nolle A, Zwart R, Boerema AS, van Haastert ES, Strijkstra AM, Hoozemans JJ, Scheper W (2014). The unfolded protein response mediates reversible tau phosphorylation induced by metabolic stress. Cell Death Dis.

[181] Macedo B, Batista AR, Ferreira N, Almeida MR, Saraiva MJ (2008). Anti-apoptotic treatment reduces transthyretin deposition in a transgenic mouse model of familial amyloidotic polyneuropathy. Biochim Biophys Acta.

[182] Zattoni M, Legname G (2021). Tackling prion diseases: a review of the patent landscape. Expert Opin Ther Pat.

[183] Cortez LM, Campeau J, Norman G, Kalayil M, Van der Merwe J, McKenzie D, Sim VL (2015). Bile acids reduce Prion Conversion, reduce neuronal loss, and Prolong Male Survival in Models of Prion Disease. J Virol.

[184] Norman G, Campeau J, Sim VL. High dose and delayed treatment with bile acids ineffective in RML prion-infected mice. Antimicrob Agents Chemother 2018, 62(8). 10.1128/AAC.00222-18 PMC6105847 29784843

