# Correction: Roles of bile acids signaling in neuromodulation under physiological and pathological conditions

**DOI:** 10.1186/s13578-023-01076-6

**Published:** 2023-07-06

**Authors:** Chen Xing, Xin Huang, Dongxue Wang, Dengjun Yu, Shaojun Hou, Haoran Cui, Lun Song

**Affiliations:** 1 Beijing Institute of Basic Medical Sciences, Taiping Road #27, Beijing, 100850 China; 2 College of Pharmacy, Jiamusi University, Jiamusi, 154007 China; 3 Anhui Medical University, Hefei, 230032 China

**Correction: Cell & Bioscience (2023) 13:106** 10.1186/s13578-023-01053-z

In this article [1] the author’s name Lun Song was incorrectly written as Lung Song. This has been corrected in this correction.

## References

[1] Xing C, Huang X, Wang D, Yu D, Hou S, Cui H, Song L (2023). Roles of bile acids signaling in neuromodulation under physiological and pathological conditions. Cell Biosci.

